# Multi-Omics Analysis of Lung Tissue Demonstrates Changes to Lipid Metabolism during Allergic Sensitization in Mice

**DOI:** 10.3390/metabo13030406

**Published:** 2023-03-09

**Authors:** Kedir N. Turi, Cole R. Michel, Jonathan Manke, Katrina A. Doenges, Nichole Reisdorph, Alison K. Bauer

**Affiliations:** 1 Department of Medicine, Vanderbilt University, Nashville, TN 37203, USA; 2 Department of Pharmaceutical Sciences, University of Colorado, Aurora, CO 80045, USA; 3 Department of Environmental and Occupational Health, University of Colorado, Aurora, CO 80045, USA

**Keywords:** multi-omics, allergy, asthma, metabolomics, lipidomics, oxylipins, transcriptomics, house dust mite

## Abstract

Allergy and asthma pathogenesis are associated with the dysregulation of metabolic pathways. To understand the effects of allergen sensitization on metabolic pathways, we conducted a multi-omics study using BALB/cJ mice sensitized to house dust mite (HDM) extract or saline. Lung tissue was used to perform untargeted metabolomics and transcriptomics while both lung tissue and plasma were used for targeted lipidomics. Following statistical comparisons, an integrated pathway analysis was conducted. Histopathological changes demonstrated an allergic response in HDM-sensitized mice. Untargeted metabolomics showed 391 lung tissue compounds were significantly different between HDM and control mice (adjusted *p* < 0.05); with most compounds mapping to glycerophospholipid and sphingolipid pathways. Several lung oxylipins, including 14-HDHA, 8-HETE, 15-HETE, 6-keto-PGF1α, and PGE2 were significantly elevated in HDM-sensitized mice (*p* < 0.05). Global gene expression analysis showed upregulated calcium channel, G protein–signaling, and mTORC1 signaling pathways. Genes related to oxylipin metabolism such as *Cox*, *Cyp450*s, and *cPla2* trended upwards. Joint analysis of metabolomics and transcriptomics supported a role for glycerophospholipid and sphingolipid metabolism following HDM sensitization. Collectively, our multi-omics results linked decreased glycerophospholipid and sphingolipid compounds and increased oxylipins with allergic sensitization; concurrent upregulation of associated gene pathways supports a role for bioactive lipids in the pathogenesis of allergy and asthma.

## 1. Introduction

Asthma is characterized by airway hyper-responsiveness, mucus hypersecretion, infiltration of the airway by eosinophils and type 2 (T2) immune response, and airway remodeling [1,2]. Evidence suggests that cytokine imbalance and metabolic perturbance are responsible for the inflammation and tissue damage resulting from asthma [3]. In addition to increased inflammation, both systemically and locally in the lung, this perturbance results in increased oxidative stress, decreased antioxidants, and increased inflammatory cytokine markers [4,5,6]. However, our understanding of allergic asthma etiology and biological mechanisms is incomplete. This is partly due to the invasive nature of lung sampling techniques, which is a major impediment in developing prophylaxis and treatment for asthma. 

Experimental animal models were developed to mimic the clinical symptoms and pathological sequalae of asthma to overcome the challenges with studying relevant human tissue. The mouse model of house dust mite (HDM)-induced allergic airways disease mimics many of the features of human asthma symptoms, including airway hyperreactivity and airway inflammation, and is increasingly used to elucidate asthma/allergic airways pathology and to evaluate new therapeutic agents [7]. However, allergic airways/asthma etiology and biological mechanisms in this experimental model were not well characterized. 

The application of high throughput ‘omics’ approaches to both human studies and animal models has shown great potential in identifying pathological mechanisms and biomarkers of asthma. For example, metabolomics measures a variety of small molecules that are part of a biological system and have potential to capture the cellular response to past and present exposures relevant to asthma etiology [8,9,10]. Since lung tissue provides an integrated, multi-cellular platform, a metabolomic investigation utilizing lung tissues may better illustrate the etiology of allergic airways/asthma and will have a significant translational value compared to those performed in isolated cells or in vitro systems [11]. Metabolomic analysis of bronchoalveolar lavage fluid (BALF) [12,13] and lung tissue [14] in sensitized mice and BALF and sputum of patients with allergic asthma [15,16] have uncovered a panel of potential allergic airway-related metabolic biomarkers and pathways. However, most of these studies did not take a multiplatform and systems approach and, hence, may have missed important relationships. 

To date, various ‘omics’ approaches increasingly implicated lipids as biomarkers for the pathogenesis and severity of asthma symptoms [17,18,19,20]. Specifically, excessive oxidative stress and its endogenous and exogenous reactive oxygen and nitrogen species, decreased activities of antioxidants, and increased production of bioactive lipids that are synthesized from arachidonic acid (AA) are all associated with airway inflammation and, consequently, with allergic asthma and its severity [21,22,23,24]. Conversely, the roles of endogenous bioactive oxylipins derived from eicosapentaenoic acid (EPA) and docosahexaenoic acid (DHA) as counter-regulators of inflammation and activators of resolution is still being established [25,26,27]. These bioactive lipids, termed oxylipins, are rapidly metabolized and are challenging to detect through untargeted metabolomics analysis. However, targeted analysis of these molecules using tandem liquid chromatography mass spectrometry (LC/MS/MS) was used extensively in the analysis of these important lipid molecules [17,28,29].

Furthermore, since genetics plays a major role in regulation of the metabolome [30] and asthma etiology, integrating metabolomics with gene expression data enhances the potential to unravel relevant pathways in diseases with gene-X-environmental etiological components such as asthma and allergic airways [31,32]. Overlaying molecular pathways based on metabolomics and gene expression data help to extract more insightful and comprehensive snapshots of biological systems and molecular processes in the etiology of asthma. Therefore, the aim of this study is to understand the upstream molecular processes underlying allergic asthma etiology in HDM-induced allergic mice using a multi-omics approach that comprises untargeted metabolomics, targeted analysis of oxylipins, and transcriptomics of lung tissue. This integrated analysis of metabolomics, lipidomics, and gene expression data provides additional insight into novel links between metabolic, immune, and neuronal signaling pathways triggered by HDM sensitization which can be investigated for possible intervention targets in future studies. 

## 2. Materials and Methods

### 2.1. Animals 

Six-week-old male BALB/cJ (BALB) mice were obtained from Jackson Laboratories (Bar Harbor, ME). This strain was chosen because it is Th_2_-dominant and commonly used in lung allergic airway models due to their classic allergic responses, including increased Th_2_-immune responses, eosinophilia, and airway hyperresponsiveness [33,34]. The mouse numbers used for each endpoint were based on animal requirements for significance in previous studies [35,36] as well as a power analysis. All mice were maintained on an ovalbumin (OVA)-free Teklad diet (Envigo). Mice were acclimated for one week prior to sensitization. Studies were conducted under a protocol (#01031) approved by the Institutional Animal Care and Use Committee at University of Colorado Anschutz Medical Campus (Aurora, CO).

### 2.2. HDM Sensitization

**Figure 1:**
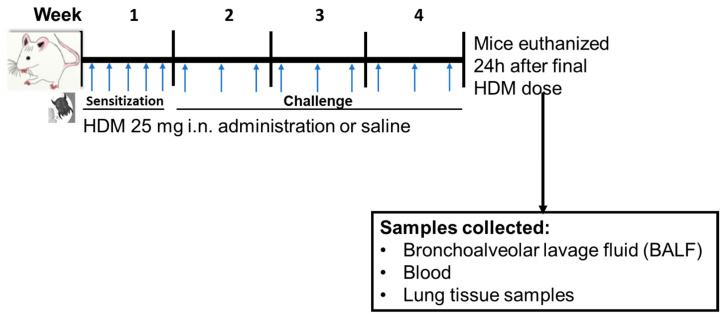
Experimental design for mice house dust mite (HDM) sensitization and challenge and sample collections. Mice were sensitized (week 1) or challenged (weeks 2–4) with HDM or saline (control) followed by euthanasia 24 h following final dose. i.n. = internasal. The blue arrow indicates timing of HDM or saline administration.

Mice were sensitized to sterile filtered 25 µg HDM extract (HDM, GREER Labs; in 35 µL saline) or saline through internasal (i.n.) administration for 5 days/week for week 1 and then challenged with 25 µg HDM (35 µL saline) or 35 µL saline for 3 days/week for weeks 2–4 of the experiment (Figure 1). All mice used in this study were treated at the same time and were euthanized 24 h following the final dose of HDM in week 4. The HDM sensitization dose, frequency, and duration was based on a recently published 4-week HDM-induced allergic airways inflammation mouse model [37].

### 2.3. Differential Cells Counts and Histology

Blood was collected via cardiac puncture and, following processing using EDTA tubes (Thermo Fisher Scientific, Waltham, MA 02451, USA), plasma was snap frozen. Bronchoalveolar lavage fluid (BALF) was collected with Hanks balanced salt solution (HBSS), described previously [38,39], using n = 5 mice per group. Cell differentials and total protein (reflective of lung hyperpermeability) was performed on BALF, as performed in Cho [39] and Bauer [36]. Following lung perfusion with sterile saline, these lungs were also inflation fixed (n = 5) with 10% neutral buffered formalin for 24 h, followed by processing by the University of Colorado Cancer Center (UCCC) Pathology Shared Resource. Hematoxylin and eosin (H&E), periodic acid–Schiff (PAS), and trichrome stained slides were performed for each of these mouse lungs (n = 5). In another group of mice (n = 4), non-lavaged and non-perfused lung lobes (with no lymph nodes) were divided for untargeted metabolomics, targeted lipidomics, and RNA transcriptomics assays.

### 2.4. Untargeted Metabolomics:

Lung samples (n = 4 for control and n = 4 for HDM) for untargeted metabolomics analysis were prepared as previously described [40,41,42]. Briefly, lungs were homogenized using a bead homogenizer with methanol and small molecules were extracted from 100 µL lung tissue homogenate using methyl tert-butyl ether (MTBE). Aqueous and lipid fractions were analyzed separately by liquid chromatography mass spectrometry (LC/MS) on a quadrupole time-of-flight (6545 QTOF, Agilent Technologies, Santa Clara, CA 95051, USA) mass spectrometer using published methods [13,43], except that 10 µL of the lipid fraction samples were injected on the instrument. All samples were prepared in a single batch and, therefore, no batch-to-batch quality control (QC) sample was used to control for sample preparation variance. However, all experimental lung samples were spiked with 29 labeled authentic standards and an aliquot of each sample was pooled post sample preparation to make a pooled QC sample to control for LCMS instrument variance [13,43]. Following analysis of QC data to ensure reproducibility (see Text S1 for details on quality control), metabolomics spectral data were extracted and recursively filtered, aligned, and binned using Agilent Profinder ver 10.0 SP1 and Mass Profiler Professional Ver. 15.1 (MPP, Agilent Technologies, Santa Clara, CA 95051, USA) [42]. Compounds found in at least one blank were removed. Remaining compounds were limited to those found in 75% of samples in at least one group (HDM or control). Aqueous samples were additionally limited to compounds eluting before 11.5 min since compounds eluting past this time had poor signal to noise ratios. Normalization was conducted using adjustment to total signal for all compounds not found in blanks.

Compounds were annotated by searching a custom in-house database comprised of data from authentic standards and public databases consisting of compounds from METLIN, Lipid Maps, Kyoto Encyclopedia of Genes and Genomes (KEGG), and Human Metabolome Database (HMDB), using accurate mass and isotope ratios. These compounds were designated Metabolomics Standards Initiative (MSI) level three [13]. Compounds matching in retention time and mass to compounds in the in-house database were designated MSI level one.

### 2.5. Quantitative Targeted Analysis of Oxylipins (Lipidomics)

Liquid chromatography tandem mass spectrometry (LC/MS/MS) and isotope dilution was used to quantitate 87 pro-inflammatory and pro-resolving lipids and isoprostanes in plasma and lung using a single, validated assay [44,45]. Briefly, samples were extracted with methanol followed by solid phase extraction to enrich for oxylipins. Internal standards, comprised of labeled analogs corresponding to 12 molecules of various lipid subclasses, were added prior to extraction. Samples were analyzed using a triple quadrupole mass spectrometer (QQQ 6490, Agilent Technologies, Santa Clara, CA 95051, USA) as previously described [44,45,46].

### 2.6. Global Gene Expression

The RNA preparation and sequencing was carried out at the UCCC Genomics Core. mRNA was isolated from mice lung tissue using TRI-Reagent (Sigma-Aldrich, St. Louis, MO 68178, USA) followed by Zymo-Seq Ribo Free Total RNA Library Kit for the library preparation. cRNA (1.5 μg) was used for whole-genome gene expression direct hybridization assay with mouse WG-6 v2.0 Expression Beadchip (Illumina, San Diego, CA 92122, USA), following the manufacturer’s instructions. The average reads/bases quality for all the samples in the lane was at least 88% ≥ Q30. The filtered reads distribution for all the samples in the lane ranged from approximately 11 M to 18 M clusters (22 M to 36 M paired end reads). Sequence data quality was evaluated using fastqcr, an R wrapper for freely available quality control software (FastQC) [47,48]. The software suites of Rsubread [49] and R package were used for mRNA read mapping, with the reference mice genome (GRCm38 primary assembly genome) and the feature counts function to quantify read counts.

### 2.7. Statistical Analysis

Compounds from untargeted metabolomics were assigned class hierarchy if they had a KEGG compound number provided and were annotated by compound class, subclass 1, subclass 2, subclass 3, and subclass 4; this provided several options to analyze and visualize data [50]. Changes in compounds between HDM and control mice were evaluated by multiple t-testing using a false discovery rate (FDR) of 5% and fold change for compounds were generated [50]. The number of significantly changed compounds between HDM-sensitized and control mice were counted based on the KEGG class annotation and visualized with bar plots. Similarly, compound abundance was aggregated by KEGG subclasses and compared between HDM-sensitized mice and control and visualized with boxplots [50]. Enrichment and pathway analyses were conducted for significantly different compounds. Metabolic reaction pathways were predicted to identify active and suppressed metabolic conversion based on upregulated and downregulated lipid compounds using Lipid Map’s BioPAN online software suite [51]. Metabolic network, network diffusion, and network topology and clustering analysis were conducted using combination of FELLA (R package) [52] and Cytoscape software suite [53]. Changes in oxylipins (from targeted lipidomics analysis) between HDM and control mice were evaluated by multiple t-testing using a false discovery rate (FDR) of 5%. Changes in BALF cells and protein were determined using t-tests (Graphpad Prism 9) on log transformed data, with *p* < 0.05 considered significant.

Gene expression read count data were converted into log2 counts using the rlog function in DESeq2 and plotted to assess data quality [54]. The read count data was filtered for zero counts before differential expression analysis was conducted. Differential gene expression (log2 fold changes [log2FC]) was conducted using negative binomial generalized linear equation in DESeq2, which accounted for library size and group mean and variance internally [54]. The estimated log2FC were shrunk to aid visualization and ranking of genes and *p*-valued corrected for inflation and biases using bacon package [55]. Enrichment and pathway analysis were conducted for significantly different genes using BioMart suite retrieving GO and KEGG molecular and functional knowledge databases [56]. Joint metabolomics and gene expression data pathway analysis were conducted using KEGG molecular knowledge databases to understand the potential interactions and involvement between the compounds and genes that were significantly changed between HDM-sensitized and control mice in the biological process and molecular functions. All statistical analyses were performed using R software version 4.1.1 (https://www.r-project.org accessed on 25 August 2021).

## 3. Results

### 3.1. Inflammatory Cells and Histopathology Changes in HDM-Sensitized Mice

**Figure 2:**
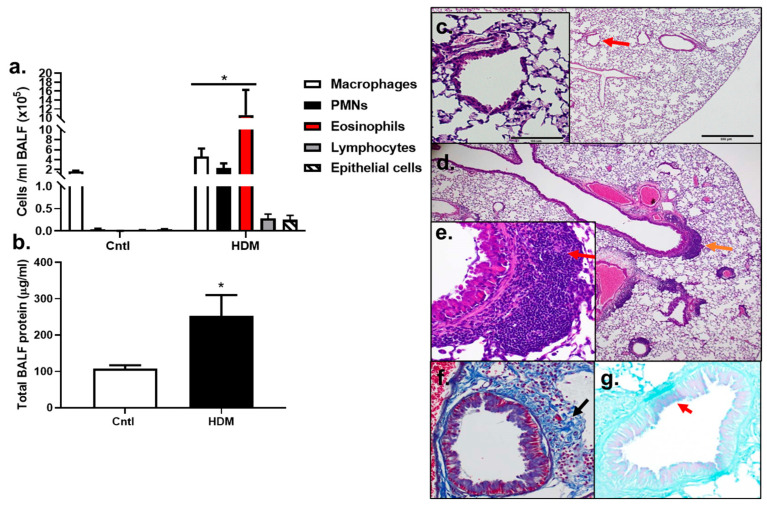
Inflammation increased in response to HDM. (**a**) BALF inflammatory cells, macrophages, polymorphonuclear leukocytes (PMNs), eosinophils, lymphocytes, and epithelial cells increased in response to the HDM allergen model in BALB mice. (**b**) Total BALF protein increased in response to HDM, indicative of lung hyperpermeability. Control n = 5; HDM n = 4. * *p* < 0.05 for HDM-sensitized mice compared to control mice. Histopathology in (**c**–**g**) show differences between control (**c**) and HDM exposed mice (**d**–**g**). (**c**) Control mouse stained with H&E at 4 and 20× magnification (inset at red arrow). (**d**) H&E of an HDM-exposed mouse at 4× (inset from region of orange arrow in (**e**) and (**e**) 20× magnification. Red arrow indicates peribronchial inflammation. (**f**) Collagen deposition in the same HDM-exposed mouse in trichome stained section (trichrome, blue; black arrow). (**g**) Goblet cell hyperplasia in PAS-stained section from the same HDM-exposed mouse (PAS staining (pinkish purple, red arrow); counterstain light green).

BALF inflammatory cell analysis demonstrated that significant increases in macrophages, PMNs, and eosinophils (*p* < 0.05) were observed in HDM-sensitized mice compared to control (Figure 2a). Lymphocytes and epithelial cells appeared to be elevated compared to control, but these increases were not significant, at *p* < 0.08 and *p* < 0.1720, respectively. Total protein was also significantly elevated in HDM-sensitized mice compared to control (*p* < 0.0232) (Figure 2b). These results demonstrate that an inflammatory response to HDM allergic sensitization occurred.

The analysis of histopathological changes in lung by H&E staining showed the typical pathological features of allergic airways and asthma in HDM-sensitized mice compared to control (Figure 2c) including inflammatory cell infiltration, specifically peribronchial inflammation (Figure 2d,e, red arrow, inset). Increased collagen deposition was also observed in the HDM-exposed mice using trichome staining (Figure 2f) and some goblet cell hyperplasia was seen in the HDM-sensitized mice compared to controls (Figure 2g). Collectively, this model had numerous similarities to human asthma and was a valuable model to use for our additional studies below.

### 3.2. Differentially Regulated Compouds (Untargeted Metabolomics) in HDM-Sensitized Mice

**Figure 3:**
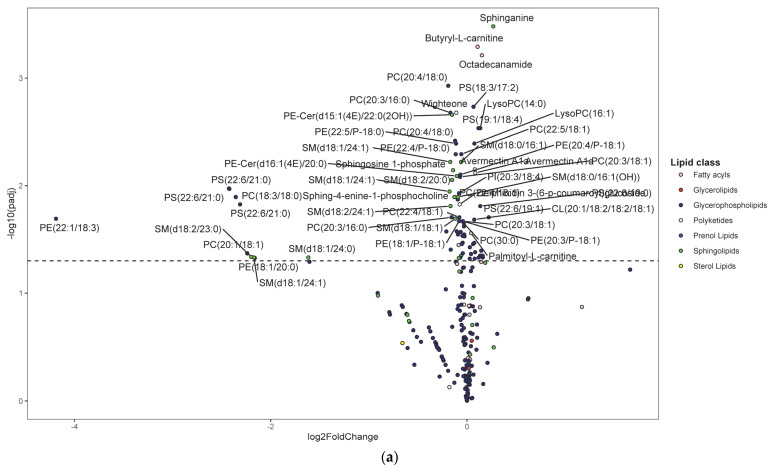

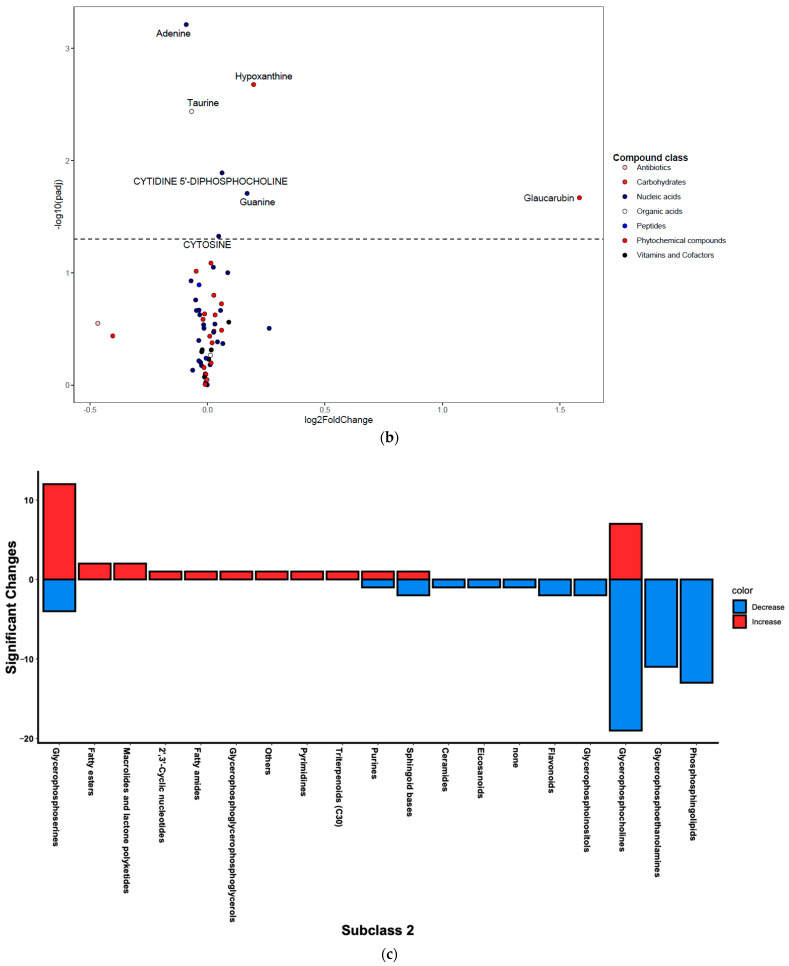
Differentially regulated compounds between house dust mite (HDM)-sensitized and control (see Table S1 for details on all significant compounds). (**a**) Differentially regulated lipid compounds between HDM-sensitized and control mice following metabolomics of lung tissue. The bubbles were colored according to the class of the compounds. The x-axis shows log 2 of the foldchange (log2 FC) and y-axis shows negative log 10 of the multiple tests adjusted *p*-values (padj). The compounds above horizontal dashed lines show significantly upregulated (log FC > 0) and downregulated (log FC < 0) lipid compounds. (**b**) Differentially regulated non-lipid compounds between house dust mite-sensitized and control mice following metabolomics of lung tissue. The bubbles were colored according to the class of the compounds. The x-axis shows log 2 of the foldchange (log2 FC) and y-axis shows negative log 10 of the multiple tests adjusted *p*-values (padj). The compounds above horizontal dashed lines show significantly upregulated (log FC > 0) and downregulated (log FC < 0) non-lipid compounds. (**c**) Histogram of significantly upregulated (red) and downregulated (blue) lung tissue compounds organized by subclass (x-axis). The number of compounds in each class (KEGG subclass 2) with significant differences between HDM-sensitized and control mice is shown.

Following untargeted metabolomics analysis of lung tissue, 1316 compounds were determined to be present in at least 75% of sample per group. Of these, 391 compounds were significantly different between the HDM and control mice (adjusted *p* < 0.05). Most of these compounds were not annotated and, therefore, were not pursued in downstream analysis and interpretations such as pathway enrichment analysis. The five most significantly upregulated compounds included 7-8-dihydro-L-biopterin, palmitoyl ethanolamide, a phosphatidylglycerol (PG [16:0/0:0]), sphinganine, and butyryl-L-carnitine (Table S1). The five most significantly downregulated compounds were Fagaramide, s-(5′-adenosyl)-l-homocysteine, adenine, PC(20:4/18:0), and PC(20:3/16:0) (Table S1). Note that these annotations were MSI level three and were hence considered putative. Changes were visualized using volcano plots for lipids (Figure 3a) and non-lipid compounds (Figure 3b).

Figure 3c shows number of significantly up- and downregulated compounds in each KEGG subclass. Most of the upregulated compounds were in diacyglycerolphosphoserines, fatty acyls (including fatty acyl carnitines and primary amides), and glycerolphosphocholines (monoacylglycerolphosphocholines and diacylglycerolphosphocholines) pathways. Note that the diacylglycerophospholipids are generally referred to as glycerophospholipids while the monoacylated forms are referred to as lyso-glycerophospholipids; however, the KEGG nomenclature was used in the current study to allow for multi-omics analysis. Enrichment analysis demonstrated that the upregulated KEGG pathways were significantly (*p*-value < 0.05) enriched in glycerophospholipid metabolism, purine metabolism, and one carbon pool by folate pathways before FDR adjustment; however, none were significant after FDR adjustment (see Figure S1 for list of pathways and Figure S2 for list of predicted enzymes). The majority of downregulated compounds were in glycerophospholipid (including diacyglycerolphosphocholines, monocyglycerolphosphocholines, diacylglycerophosphoethanolamines, and 1-Z-alkenyl,2-acyglycerolphosphocholines, as shown in Table S1), and phosphosphingolipids (including ceramide phosphoethanolamines and ceramide phosphocholines [shingomyelins]) (Figure 3c). At least one compound was changed in the following pathways: glycerophosphoinositols, flavonoids, purines, and eicosanoids (only PGD2 was significant) pathways (Figure 3c). Enrichment analysis demonstrated that the upregulated KEGG pathways were significantly (*p*-value < 0.05) enriched in sphingolipid metabolism, glycerophospholipid metabolism, and taurine and hypotaurine metabolism pathways before FDR adjustment. However, only sphingolipid metabolism pathway was significant after FDR adjustment (see Figure S1 for list of pathways and Figure S2 for list of predicted enzymes). Pathways such as glycerophosphatidylcholines, phosphatidylserines, sphingoid bases, and purines contained a mix of up- and downregulated individual compounds; the results from above were based on the direction of the majority of the compounds.

Moreover, expansion of compounds’ knowledge-based network (network diffusion) by including KEGG metabolic pathways and GO terms indicates that the dysregulated compounds were associated with inflammatory pathways (see Supplemental Text S1 and Figure S3 for detail of results). In addition, since most of the significantly altered compounds were lipids, we conducted an analysis of predicted reaction pathway for differentially abundant lipid compounds to identify most active lipid conversions in our experiment. The reaction prediction was conducted in Lipid Map’s BioPAN [51] software suite. The reaction pathway prediction showed that the most active conversion pathways were from phosphotidylcholines and phosphotidylethanolamines to phosphotidylserines and from sphingomyelins (n-acyl-sphing-4-enine-1-phosphocholine) to ceramides (n-acyl-sphing-4-enine) (Figure S4).

### 3.3. Differentially Regulated Oxylipins (Targeted Lipidomics) in HDM-Sensitized Mice

**Figure 4:**
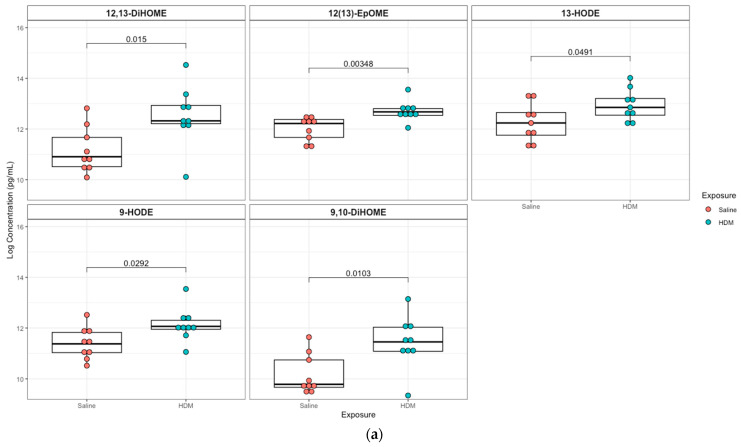

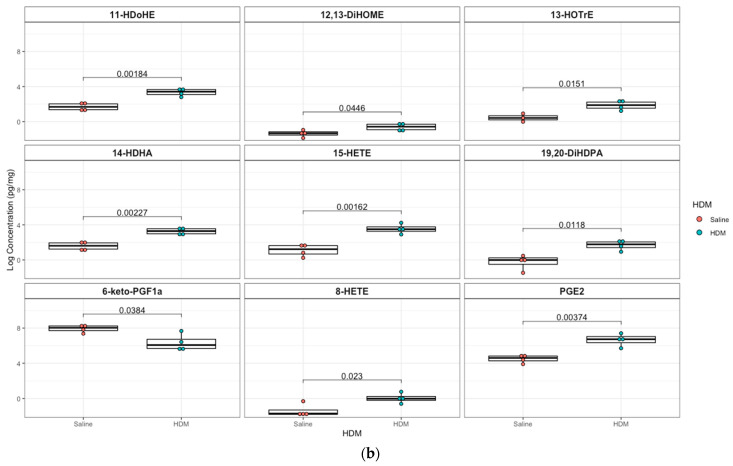
Box plots of significantly different oxylipins between house dust mite (HDM)-sensitized and control mice plasma and lung tissue. (**a**) Significantly different oxylipins in mice plasma (see Figure S5 for all oxylipins). (**b**) Significantly different oxylipins in mice lung tissue (see Figure S6 for all oxylipins). The t-test was used for statistical comparison for HDM-sensitized and control. *p*-values provided for each lipid panel were not adjusted for multiple testing.

A targeted quantitation of 87 oxylipins was conducted in plasma and lung tissue using mass spectrometry. Twenty-one plasma and twenty-eight lung lipid mediators had values above the detection limit for 80% of the samples. In plasma, 12,13-epoxyoctadecenoic acid (12,13-EpOME; *p* = 0.003) and its downstream metabolites 9,10-dihydroxy-octadecenoic acid (9,10-DiHOME; *p* = 0.01), 12,13-dihydroxy-octadecenoic acid (12,13-DiHOME; *p* = 0.02), 9-hydroxy-octadecadienoic acid (9-HODE; *p* = 0.03), and 13-hydroxy-octadecadienoic acid (13-HODE; *p* = 0.05) were significantly higher in the HDM group compared to control, but only 12,13-EpOME was significant (adjusted *p* = 0.07) after adjusting for multiple-testing using FDR at 10%. In addition, analysis of lung tissue oxylipins showed that 11-hydroxydocosahexaenoic acid (11-HDoHE, *p* = 0.002), 12,13-dihydroxy-octadecenic acid (12,13-DiHOME, *p* = 0.05), 13-hydroxyoctadecatrienoic acid (13-(S)-HOTrE; *p* = 0.02), 14-hydroxy-docosahexaenoic Acid (14-HDHA, *p* = 0.002), 8-hydroxyeicosatetraenoic Acid (8-HETE, *p* = 0.002), 15-hydroxyeicosatetraenoic Acid (15-HETE, *p* = 0.02), (19,20-DiHDPA, *p* = 0.01), 6-keto-prostaglandin F1α (6-keto-PGF1α; *p* = 0.04), prostaglandin E2 (PGE2; *p* = 0.004) were significantly elevated in the HDM-sensitized group; but none were significant after adjusting for multiple testing (Figure 4; see Figures S5 and S6 for additional lipids identified in plasma and lung samples, respectively).

### 3.4. Differentially Expressed Genes (Global Gene Expression) in HDM-Sensitized Mice

**Figure 5:**
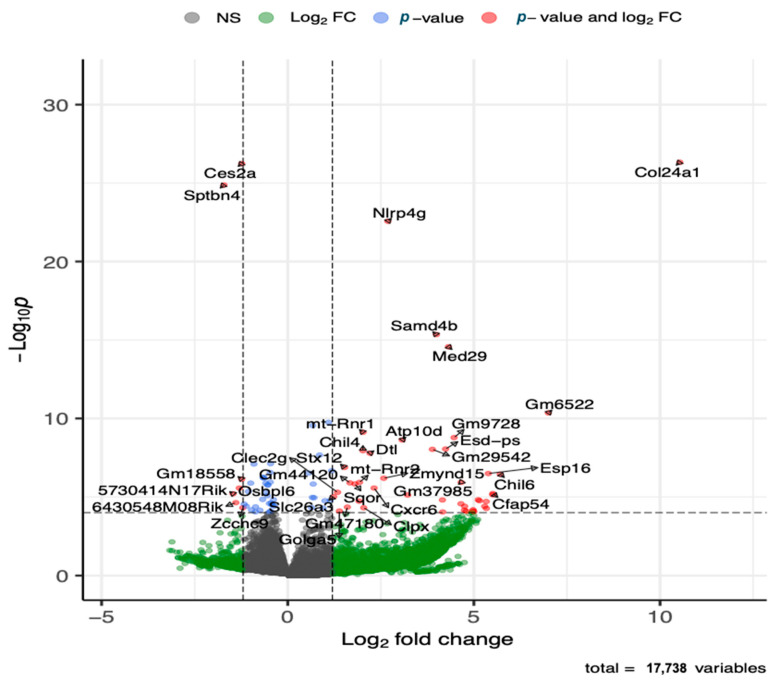
Volcano plot of differentially upregulated and downregulated genes following transcriptomics analysis of lung tissue (see Table S2 for details on all significant genes). The x-axis shows log 2 transformed fold-change and y-axis shows negative log 10 transformed *p*-value. Horizontal dashed line is a cut-off *p*-value at 0.05 adjusted for multiple testing. The two vertical dashed lines represent a fold-change window with genes to the left or right of the window at greater or less than 1.5 fold-change.

The global gene expression analysis of lung tissue showed that several genes (143 genes) were differentially expressed after adjusting for multiple testing at adjusted *p*-value of 0.05 and fold change cutoff value of 1.5, where the majority (88 genes) of the genes were upregulated (Figure 5 and Table S2). The top five upregulated genes included *Col24a*, *Nlrp4g*, *Samd4b*, *Med29*, and *Ganab*. Additional immune response genes that were upregulated in the HDM-sensitized group included *Chil4*, *Chil6*, *Cxcr6*, *Macir*, and *Clec2g*, and metabolism related upregulated genes included *Atp10d* and *Ivd*. Although not statistically significant, cytochrome c oxidase (Cox)-10, cytochrome P450 (Cyp450)-2E1, Cyp450-2j3, and cytosolic phospholipase A2 (cPla2)-g4, and elongation of very long chain fatty acids protein 4 (Elov4) were among many lipid metabolism regulation genes that were upregulated with log2 foldchange >2. The top five downregulated genes included *Ces2a*, *Sptbn4*, *Hsd17b1*, *Nrxn2,* and *Gpr137c*. Additional immune response genes that were downregulated in the HDM-sensitized mice included *Btnl4*, *Lifr*, *Atm*, and *Zcchc9*, and additional metabolism genes that were downregulated in the HDM-sensitized mice included *Mtm1*, *Ggta*, *Akt2*, *Repin1*, *Rabgap1l*, *Pask*, and *Csad.* The supportive literature for the functions of these genes are summarized in Table S3.

To summarize the biological and molecular context of significantly expressed genes (multiple testing adjusted *p*-value < 0.05), we conducted enrichment analyses using the GO knowledge database. The GO biological process enrichment analysis showed that the following were the most enriched biological processes pathways for significantly upregulated genes in the HDM-sensitized mice compared to control: regulation of cardiac muscle (cell contraction) by regulation of the release and transportation of sequestered calcium ion, metal ion transport, collagen fibril organization, and calcium-mediated signaling. Regulation of vesicle fusion, central nervous system neuron axonogenesis, regulation of glycogen biosynthetic process, regulation of torc1 signaling, and regulation of B-cell proliferation were the five most enriched biological processes for downregulated genes in HDM-sensitized mice compared to control (Table 1).

Similarly, the GO molecular function enrichment analysis of significantly expressed genes showed that voltage-gated calcium channel activity involved in muscle cell action potential, benzodiazepine receptor binder, solute and sodium bicarbonate symporter activity, oncostatin M receptor activity, leukemia inhibitory factor receptor activity, and g protein-coupled serotonin receptor binding were the five most enriched pathways for upregulated genes. Testosterone dehydrogenase [nad] activity, phosphatidylinositol-3,5-bisphospate 3-phosphatase activity, neuroligin family protein binding, ccr5 chemokine receptor binding, and annealing activity were the five most downregulated molecular functions in downregulated genes in HDM compared to control mice (Table 2).

### 3.5. Joint Pathways of Differentially Regulated Metabolic Compounds and Differentially Expressed Genes in HDM-Sensitized Mice

A joint KEGG pathways enrichment analysis was conducted for significantly changed compounds and genes between HDM-sensitized and control mice to understand the comprehensive metabolic pathway dysregulation that we hypothesized as occurring with allergic responses to HDM. Accordingly, glycerophospholipid and sphingolipid metabolism were the most jointly enriched KEGG molecular pathways (Figure S7) affected by HDM sensitization. In addition, joint enrichment of insulin secretion, cholinergic synapse, adrenergic signaling in cardiomyocytes, choline metabolism, calcium signaling, and apelin signaling pathways was seen. However, only glycerophospholipid metabolism and sphingolipid metabolism were significant after FDR adjustment. Genes that were involved in regulation of lipid phosphorylation, including *Mtm1*, *Ggta*, and *Mtmr4*, were downregulated.

## 4. Discussion

This study aimed to better understand the effects of allergen sensitization on metabolic and immune signaling pathways by comparing small molecule and gene expression differences between HDM-sensitized and control mice using a multi-omics approach. Overall, our findings confirmed previous results by our group [13] and others that demonstrated the dysregulation of purine, glycerophospholipid, and sphingolipid metabolism, as well as the AA and LA oxidation products 9-HODE and 12,13-EpOME (and its downstream metabolites) in allergen-sensitized mice. Furthermore, the current study demonstrated that additional signaling pathways, such as cardiolipin and insulin secretion pathways, were dysregulated in allergen-sensitized mice.

Our results from untargeted metabolomics indicated that the majority of dysregulated compounds were glycerophospholipids, similar to previous reports [12,57]. Compounds within the class of phosphosphingolipids and certain sub-classes of glycerophospholipids, including glycerophosphatidylcholines, glycerophosphatidylethanolamines, and glycerophosphatidylinositols were downregulated, whereas diacyglycerolphosphoserines and glycerophosphate were upregulated. These compounds are all part of the highly interconnected glycerophospholipid pathway, whereby glycerophosphate (1,2-diacyl-sn-glycerol-3-phosphate) acts as a precursor to the glycerophospholipids through the intermediates phosphatidic acid, sn-1,2-diacylglycerol, and/or CDP-diacylglycerol. Similarly, the diacylglycerophospholipids are metabolized to corresponding monoacylated forms and can also be used to generate phosphatidic acid. It is possible that glycerophosphatidylcholines, glycerophosphatidylethanolamines, and glycerophosphatidylinositols were depleted to meet cellular demands during inflammation. For example, the prediction model based on our data shows that, in HDM-sensitized mice, phosphatidylcholines and phosphatidylethanolamines were actively converting to phosphatidylserines, whereas sphingomyelins were actively converting to ceramides. Under normal physiological conditions, significant amounts of phosphatidylserines turn over to form phosphatidylethanolamines [58]; however, the conversion may be reversed in disease-like conditions, such as allergic airways.

The importance of phospholipids in asthma pathogenesis were previously described [59]. For example, phosphatidylcholines were decreased in asthmatic lungs [12,57] and children with risk allelles in the 17q12-21 genetic region have decreased sphingolipid synthesis [60]. This is consistent with our finding that glycerophosphatidylcholine levels were decreased in sensitized mouse lung. Similarly, decreased levels of glycerophosphatidylethanolamines and glycerophosphatidylinositols and increased diacyglycerolphosphoserines in plasma samples among asthmatic patients were previously observed [61]. Although not directly measured in the current study, the observed downregulation of glycerophosphatidylcholines in lung following allergic sensitization may be interpreted to reflect metabolism of EPA or DHA via cPLA2 to more pro-resolving and/or anti-inflammatory oxylipins. In addition, glycerophosphotidylcholines have roles in anti-inflammatory mechanisms including suppression of TNF production in macrophages and interference with pro-inflammatory cytokines secreted by phagocytes [62,63,64]. Finally, the precursor of diacyglycerolphosphoserines, phosphatidylserine, may play an important role in T2 immune response induction and airway hyperreactivity [65]. Thus, by extension, upregulated diacyglycerolphosphoserines in HDM-sensitized mice in our study is consistent with allergic asthma pathogenesis.

**Figure 6:**
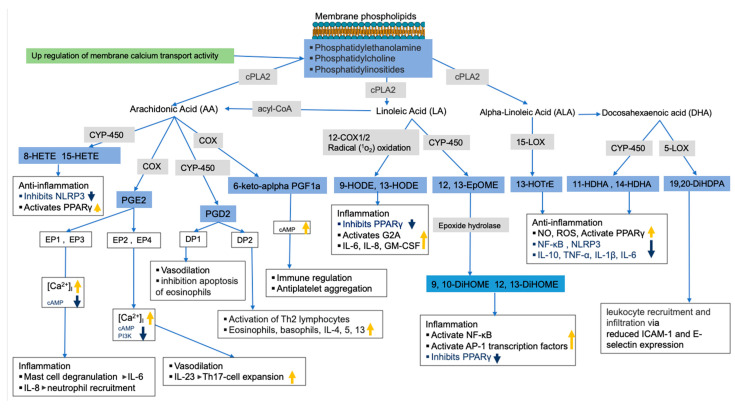
Allergy sensitization lipid regulation pathway. Pathways were constructed using results from current study (in blue boxes for metabolites and green for gene pathway) and previously published studies. Gray boxes show enzymes involved in bioactive lipids synthesis (related genes were upregulated, but not significant). The thick orange and blue arrows show the direction of the signaling pathways’ regulations based on previous studies.

As previously mentioned, membrane glycerophospholipids such as glycerophosphoserine contain fatty acids such as AA, LA, EPA, and DHA, which are precursors of bioactive lipid mediators such as oxylipins and endocannabinoids [66,67,68,69]. While the untargeted metabolomics analysis of lung tissue showed that most glycerophospholipids were decreased in HDM-sensitized mice, the targeted analysis of oxylipins in the same tissue led to an increase in these bioactive lipids in HDM-sensitized mice. In addition, we observed upregulated but statistically insignificant genes related to the release and metabolism of membrane fatty acids to bioactive lipids such as *Cox*, *Cyp450*s, and *cPla2* as well as calcium binding regulator genes. Thus, based on the observed depletion of glycerophospholipids and increased oxylipins, along with the upregulation of related genes, we speculate that allergic sensitization results in a conversion of membrane lipids to oxylipins. In support of this, we observed increases in AA-derived 8-HETE, 15-HETE, PGE_2_, and 6-keto-α-prostaglandin F_1α_ (6keto-α-PF_1α_), which are known for both pro-inflammatory and anti-inflammatory properties often depending on receptor and tissue type (see Figure 6 for illustration). For example, PGE_2_ was shown to increase mast cell degranulation and IL-6 production, IL-8-induced neutrophil recruitment, vasodilation, among others [70]. 6-keto-α-prostaglandin F_1α_ is a less potent and stable derivative of prostacyclin I_2_ (PGI_2_) known to serve as antiplatelet aggregation though the upregulation of cAMP activities [71,72] and immune regulators [73].

Increased LA oxidation products such as 9-HODE and 12,13-EpOME (and its downstream metabolites 9,10-DiHOME and 12,13-DiHOME) were also observed in HDM-sensitized mice, which is consistent with their known pro-inflammatory roles. For example, 9-HODE plays a role in inflammation by activating the G protein coupled receptor 132 (G2A), inhibiting the peroxisome proliferator-activated receptor γ (PPARγ), and increasing production of inflammatory cytokines such as IL-6, IL-8, and GM-CSF [74]. Similarly, 12,13-EpOME (and its downstream metabolites) increased inflammation by activating NF-κB and AP-1 transcription factors and inhibiting PPARγ [75,76,77]. Generally, increased LA oxidation products associate with features of severe airway obstruction, lung remodeling, increase in epithelial stress related to pro-inflammatory cytokines and airway neutrophilia in mice [78]. A potential future intervention study could inhibit LA oxidation to determine if the inflammatory response to allergic challenges and symptoms of asthma improve.

Alpha linoleic acid (αLA) and DHA-derived oxylipins known for their pro-resolving characteristics were also increased in sensitized mice. For example, 13-(S)-HOTrE, which is derived from αLA, exhibited anti-inflammation properties through inhibition of NF-κB and NLRP3 and through activation of PPARγ [79,80]. Likewise, DHA-derived 19,20-DiHDPA was shown to modulate leukocyte recruitment and infiltration via reduced ICAM-1 and E-selectin expression in endothelial cells [81,82]. The increase in these oxylipins during HDM sensitization suggests a counterbalance exists between pro-inflammatory and pro-resolving oxylipins.

Moreover, lipids are also known to modulate the activity of voltage-gated ion channels including calcium channel and G protein–signaling mechanisms [83,84]. As previously mentioned, phospholipases such as cPLA2 hydrolyze glycerophospholipids by binding to membrane G-protein-coupled receptors and releasing free fatty acids, such as AA and LA, from the membrane [85,86]. Our pathway analysis of differentially expressed genes confirmed the involvement of ion channels and bioactive lipids signaling pathways during HDM sensitization. We observed the upregulation of pathways such as release and transportation of sequestered calcium ion release, sequestration and transportation, metal ion transport, collagen fibril organization, and calcium mediated signaling in sensitized mice. The increase in Ca^2+^ in cytoplasm is associated with asthma pathology including activation of respiratory smooth muscle, mast cells, vagal reflex stimulation, secretion of the airway submucous glands, and chemotaxis of eosinophils [87,88]. Based on combined results from gene expression and metabolomics data, we speculate that one effect of Ca^2+^ on asthma pathology may be through modulation of lipid signaling pathways.

This study used multi-omics to comprehensively illustrate molecular pathways involved during allergic sensitization in an animal model; however, it did have several limitations. First, the experiment was only conducted in male mice, which limited our ability to explore sex differences. Second, the lung tissue sample had to be rationed for histology, untargeted metabolomics, targeted lipidomics, and global gene expression data generation from different mice; thus, the metabolomics and gene expression data were not generated from the same animal. Third, the lung lobes for the omics studies were not perfused and, therefore, may have contained blood. While this could have impacted the responses observed, the filtering of compounds during data processing and the application of MTC during data analysis minimized the potential for artifacts related to blood contamination. In addition, previous studies utilized non-perfused tissues for molecular and omics research since immune cells infiltrate the lung via the blood and may contribute to the inflammatory response observed. Finally, the majority of our metabolomics data were annotated to an MSI level three and may have included mis-annotations, which can affect downstream pathway analysis.

## 5. Conclusions

The metabolomics data demonstrated downregulation of glycerophosphatidylcholines, glycerophosphatidylethanolamines, phosphosphingolipids, and glycerophosphoinositols, whereas several diacyglycerolphosphoserines and glycerophosphate were upregulated in HDM-sensitized mice. A focused analysis of oxylipins from lung tissue and plasma showed consistent results with previous studies linking decreased glycerophospholipid and sphingolipid compounds with increased bronchoreactivity and increased 12,13-EpOME (and its downstream compounds) and prostaglandins with allergic sensitization. The global gene expression analysis added another layer by linking differential changes in bioactive lipids to up- and downregulated signaling pathways such as calcium ion channels and G protein–signaling. For example, calcium channels and G protein–signaling modulate cPLA_2_ for the release of membrane lipids, which are substrates for downstream bioactive lipids. In summary, our study, using multi-omic analyses of mouse lung tissue during HDM sensitization, provided additional insight into molecular cascades during allergic sensitization including supporting known roles for AA metabolism.

## Figures and Tables

**Table 1:** Significantly upregulated and downregulated Go biological processes pathways following gene expression analysis of HDM-sensitized and control mice lung tissue. The odds ratio was defined as the ratio of the proportion of a GO term in upregulated and downregulated genes to the proportion of this GO term in all diatom genes.

Term	Odds Ratio	Genes	Regulation
Positive regulation of vesicle fusion	142.41	Akt2, Doc2b	Down
Regulation of vesicle fusion	101.71	Akt2, Doc2b	Down
Central nervous system neuron axonogenesis	64.71	Chrnb2, Sptbn4	Down
Central nervous system projection neuron axonogenesis	64.71	Chrnb2, Sptbn4	Down
Regulation of glycogen biosynthetic process	29.64	Akt2, Pask	Down
Positive regulation of organelle organization	22.94	Akt2, Doc2b	Down
Regulation of TORC1 signaling	21.55	Atm, Gpr137c	Down
Regulation of B cell proliferation	16.15	Chrnb2, Atm	Down
Organic hydroxy compound biosynthetic process	14.80	Osbpl6, Hsd17b1	Down
Regulation of calcium ion transmembrane transport via high voltage-gated calcium channel	52.66	Camk2d, Cacna2d1	Up
Regulation of cardiac muscle contraction by regulation of the release of sequestered calcium ion	31.59	Ryr2, Camk2d	Up
Cardiac muscle cell contraction	27.87	Camk2d, Cacna2d1	Up
Regulation of cardiac muscle contraction by calcium ion signaling	24.93	Ryr2, Camk2d	Up
Calcium ion transport into cytosol	24.93	Ryr2, Cacna2d1	Up
Calcium-mediated signaling using intracellular calcium source	24.93	Ryr2, Stimate	Up
Regulation of release of sequestered calcium ion into cytosol by sarcoplasmic reticulum	22.55	Ryr2, Camk2d	Up
Regulation of calcium ion transmembrane transport	22.55	Camk2d, Cacna2d1	Up
Regulation of cardiac muscle cell action potential	20.59	Ryr2, Camk2d	Up
Regulation of cardiac muscle cell contraction	18.94	Ryr2, Camk2d	Up
Cytosolic calcium ion transport	18.21	Ryr2, Cacna2d1	Up
Ion homeostasis	16.91	Slc4a8, Camk2d	Up
Cardiac muscle cell action potential involved in contraction	16.33	Ryr2, Cacna2d1	Up
Cardiac muscle contraction	14.79	Ryr2, Camk2d	Up
Metal ion transport	8.43	Ryr2, Cacna2d1, Cdh23	Up
Collagen fibril organization	8.33	Col24a1, Col11a2, Col19a1	Up
Calcium-mediated signaling	7.23	Ryr2, Stimate, Cxcr6	Up

**Table 2:** Significantly upregulated and downregulated GO Molecular processes pathways following gene expression analysis of HDM-sensitized and control mice lung tissue. The odds ratio was defined as the ratio of the proportion of a GO term in upregulated and downregulated genes to the proportion of this GO term in all diatom genes.

Term	Odds.Ratio	Genes	Regulation
Testosterone dehydrogenase [NAD(P)] activity	87.45	Hsd17b1	Down
Chromatin insulator sequence binding	87.45	Repin1	Down
RNA strand annealing activity	87.45	Eif4b	Down
Neuroligin family protein binding	87.45	Nrxn2	Down
CCR5 chemokine receptor binding	87.45	Cnih4	Down
Phosphatidylinositol-3,5-bisphosphate 3-phosphatase activity	87.45	Mtm1	Down
Annealing activity	87.45	Eif4b	Down
Oncostatin M receptor activity	69.95	Lifr	Down
Leukemia inhibitory factor receptor activity	69.95	Lifr	Down
Mannosyl-oligosaccharide 1,2-alpha-mannosidase activity	58.29	Man1b1	Down
Mannosyl-oligosaccharide mannosidase activity	58.29	Man1b1	Down
Phosphatidylinositol-3,5-bisphosphate phosphatase activity	58.29	Mtm1	Down
Estradiol 17-beta-dehydrogenase activity	49.96	Hsd17b1	Down
Ciliary neurotrophic factor receptor activity	49.96	Lifr	Down
Ciliary neurotrophic factor receptor binding	43.71	Lifr	Down
1-phosphatidylinositol-3-kinase activity	38.86	Atm	Down
Acetylcholine-gated cation-selective channel activity	34.97	Chrnb2	Down
Phosphatidylinositol 3-kinase activity	31.79	Atm	Down
Water channel activity	29.14	Aqp6	Down
Phosphatidylinositol kinase activity	24.97	Atm	Down
Water transmembrane transporter activity	24.97	Aqp6	Down
Phosphatidylinositol-3-phosphatase activity	24.97	Mtm1	Down
Nuclear import signal receptor activity	23.31	Ipo4	Down
Ribosomal small subunit binding	21.85	Eif4b	Down
Phosphatidylinositol monophosphate phosphatase activity	21.85	Mtm1	Down
phosphatidylinositol binding	7.88	Pask, Mtm1	Down
Benzodiazepine receptor binding	58.56	Tspoap1	Up
Voltage-gated calcium channel activity involved in cardiac muscle cell action potential	58.56	Cacna2d1	Up
Oncostatin M receptor activity	46.84	Prlr	Up
Sodium:bicarbonate symporter activity	46.84	Slc4a8	Up
Solute:bicarbonate symporter activity	46.84	Slc4a8	Up
Alpha-glucosidase activity	46.84	Ganab	Up
Leukemia inhibitory factor receptor activity	46.84	Prlr	Up
G protein-coupled serotonin receptor binding	46.84	Gna11	Up
Bicarbonate transmembrane transporter activity	36.45	Slc4a8, Slc26a3	Up
Chloride transmembrane transporter activity	33.84	Slc4a8, Slc26a3	Up
Ciliary neurotrophic factor receptor activity	33.46	Prlr	Up
Sodium channel inhibitor activity	33.46	Camk2d	Up
Glucosidase activity	33.46	Ganab	Up
Ciliary neurotrophic factor receptor binding	29.27	Prlr	Up
Oxalate transmembrane transporter activity	29.27	Slc26a3	Up
Acyl-CoA dehydrogenase activity	29.27	Ivd	Up
Lys63-specific deubiquitinase activity	26.02	Otud4	Up
Intracellular ligand-gated ion channel activity	23.42	Ryr2	Up
Small GTPase binding	4.15	Unc13b, Dock4, Golga5	Up

## Data Availability

Metabolomics data are available as supplement to this manuscript.

## Supplementary Materials

The following supporting information can be downloaded at: https://www.mdpi.com/article/10.3390/metabo13030406/s1, Figure S1: Dot plot showing pathway enrichment analysis of up-(a) and down-(b) regulated compounds in house dust mite (HDM)-sensitized mice experiment. Figure S2: Dot plot showing predicted enzyme dysregulation based on enrichment analysis of up- and down- (b) regulated compounds found to be significant in house dust mite (HDM)-sensitized experiment. Figure S3: Molecular network expansions (diffusion method) applied to metabolic signaling pathways. Figure S4: Lipid reaction prediction: the most active lipid conversion pathway. Figure S5: Box plots of plasma bioactive lipids comparing house dust mite (HDM)-sensitized to control mice. Figure S6: Box plots of lung tissue bioactive lipids comparing house dust mite (HDM)-sensitized to control mice. Figure S7: Joint metabolomics and gene expression data pathway enrichment analysis. Table S1: Significantly up- and downregulated compounds in house dust mite (HDM)-sensitized mice compared to control mice. Table S2: Significantly up- and downregulated genes in house dust mite (HDM)-sensitized mice compared to control mice. Table S3: Relevant genes function literature review. Text S1: Untargeted metabolomics quality control and metabolic knowledge-based network. Data S1: Lung Metabolomics Lipid Positive. Data S2: Lung Metabolomics Aqueous Positive.
