# Supplementary material for: Multi-Omics Analysis of Lung Tissue Demonstrates Changes to Lipid Metabolism during Allergic Sensitization in Mice
Source: Metabolites. 2023 Mar 9;13(3):406. doi: 10.3390/metabo13030406 (PMC10054742; doi:10.3390/metabo13030406)
Supplement: Supplementary file 1 [file metabolites-13-00406-s001.zip › metabolites-2223757-supplementary materials.pdf]

Supplementary Materials

Text S1: Supplemental text for Untargeted metabolomics quality control and Metabolic Knowledge-based network

1. **Untargeted metabolomics quality control:**

Quality control (QC) included analysis of samples pooled from extracted lung homogenate samples. Twenty-nine (29) stable isotope labelled authentic standards, 14 hydrophobic, and 15 hydrophilic were spiked into experimental samples[1,2]. An aliquot of all experimental samples was combined into a pooled QC sample and this sample was used to monitor instrumental variations. There were no QC samples prepared alongside experimental samples, as this was a small pilot study and there was only one day of sample preparation needed.

Analysis of authentic standards in pooled QC samples was performed by generating extracted ion chromatograms (EIC) using MassHunter Qualitative Analysis software version B.07 (Agilent Technologies, Santa Clara, CA) to determine if a set of samples required re-injection. Standards in QC samples were evaluated visually for reproducible peak shape and height, as well as retention time (RT) by overlaying EICs. If there was an outlier (e.g., a QC sample had multiple standards that were not reproducible within the LCMS batch), the experimental samples bracketed by that QC sample were re-injected. Once all samples were run, the signal height for each standard was analyzed in Profinder version 10.0 SP1 (Agilent Technologies) using batch targeted feature extraction. The coefficient of variation (%CV) for each standard was calculated for pooled QC and experimental samples (QC and experimental samples were kept separate for %CV calculations). For this experiment, %CVs for the authentic standards were ≤10% for QC samples, ≤30% for lipid experimental samples and ≤20% for aqueous experimental samples. This is typical for this method and sample type. Variation was expected to be higher in experimental samples compared to QC samples due to biological variability stemming from experimental conditions. Note that if %CVs had been outside of these ranges, results would have been further investigated to determine if samples required re-analysis and/or if outliers existed and should be excluded from further analysis.

For pooled QC samples, total ion chromatograms (TICs) were overlaid and compared to ensure chromatography was reproducible. Pooled QC samples are also compared to experimental samples using principal components analysis (PCA) to determine if outliers were present. For the current study, overlaid QCs demonstrated acceptable reproducibility and no outliers were seen with PCA. In addition to the above measures, UHPLC pressure was monitored and was +/- 1% across all runs.

2. **Metabolic knowledge-based network**

Expansion of compounds' knowledge-based network (network diffusion) by including KEGG metabolic pathways and GO terms indicates that the dysregulated compounds were associated with inflammatory pathways (Figure S3). The analysis showed that dysregulation in lipid metabolic pathways were implicated in the neurotrophine signaling pathway, adipocytokine signaling pathway, AGE-RAGE signaling pathway in diabetes complication, and leishmaniasis pathway via n-acyl-sphingosine [Cer(d18:1/24:1)] which was significantly decreased in HDM-sensitized mice; leishmaniasis and systematic lupus erythematosus pathways via phosphatidylserines [PS(22:6/19:0), PS(17:2/22:4), PS(22:6/17:1), PS(19:1/18:4), PS(22:6/19:1), and PS(18:3/17:2)] which were increased in HDM-sensitized mice; cardiolipin synthase via cardiolipin/1,3-diphosphatidyl-sn-glycerol [CL(20:1/18:2/18:2/18:1)] and phosphatidylglycerol [PG(16:0/0:0)], which were increased in HDM-sensitized mice; and autophagy via phosphatidylethanolamines [(PE(22:4/P-18:0), PE(20:4/P-18:1), PE(22:1/18:3), PE(18:1/P-18:1), PE(20:3/P-18:1), PE(P-38:4), PE(18:2/P-16:0), PE(18:1/18:0), PE(18:1/P-16:0), and PE(18:1/20:0)] which were decreased in HDM-sensitized mice.

Supplemental figure label

Figure S1. Dot plot showing pathway enrichment analysis of up- and downregulated compounds in house dust mite (HDM)-sensitized mice experiment. Enrichment ratio was calculated by dividing the number of hits within a particular metabolic pathway by the expected number of number of hits. Dot size increased with enrichment ratio and darker dot color indicates lower p-value.

Figure S2. Dot plot showing predicted enzyme dysregulation based on enrichment analysis of up- (a) and down- (b) regulated compounds found to be significant in-house dust mite (HDM)-sensitized versus control mice. Enrichment ratio was calculated by dividing the number of hits within a particular metabolic pathway by the expected number of number of hits. Dot size increased with enrichment ratio and darker dot color indicates lower p-value.

Figure S3. Molecular network expansions (diffusion method) to metabolic signaling pathways. Yellow eclipses are input compounds that were significantly altered in house dust mite (HDM)-sensitized mice in our experiment. Blue triangles are KEGG metabolic modules. The green hexagons are enzymes of metabolic reactions. Pink rectangles are metabolic reactions. The red diamonds are metabolic and signaling pathways relevant to the compounds and possibly to the allergy and asthma pathogenesis signaling pathways.

Figure S4. Lipid reaction prediction: the most active lipid conversion pathway. The reaction pathway was based on the Lipid Map's BioPen online software suite. Z-score is a cumulative distribution conversion of p-value of comparison between treatment and control subtracted from one (e.g., 1 – X). The Z-score is then used to predict whether a particular reaction/pathway is significantly ($p < 0.05$, the corresponding Z-score is computed to be >1.645.) changing between control and treated conditions. Consequently, if a pathway has Z-score > 1.645, then a pathway is classified as active, or non-active otherwise. See reference in the text for detail.

Figure S5. Box plots of plasma bioactive lipids comparing house dust mite (HDM)-sensitized to control mice. The t-test was used for comparison between HDM-sensitized and control mice. Unadjusted p-values are provided for each lipid.

Figure S6. Box plots of lung tissue bioactive lipids comparing house dust mite (HDM)-sensitized to control mice. The t-test was used for comparison between HDM-sensitized and control mice. Unadjusted p-values are provided for each lipid.

Figure S7. Joint metabolomics and gene expression data pathway enrichment analysis. The x-axis shows the impact of the pathways and y-axis shows the negative log 10 transformed p-values of the pathways. The size of the bubble indicates the number of hits from experimental data in a particular pathway. Pathway impact is calculated by adding up the importance measures of each of the matched compounds and then dividing by the sum of the importance measures of all compounds in each pathway.

Supplemental Tables

Table S1. Significantly up- and downregulated compounds in house dust mite (HDM)-sensitized mice compared to control mice. This list contains only annotated compounds. Compounds in all capital letters with ** denote those that matched to the in-house database comprised of authentic standards and are identified at MSI level 1.

Metabolite	log2 Fold Change	P-adj	KEGG	Class	Subclass 1	Subclass 2	Subclass 3	Regulation
Fagaramide	-0.26	1.1E-04	C10455	NA	NA	NA	NA	Down
S-(5′-ADENOSYL)-L-HOMOCYSTEINE	-0.09	3.8E-04	C00021	NA	NA	NA	NA	Down
Adenine	-0.09	6.1E-04	C00147	Nucleic acids	Bases	Purines	none	Down
PC(20:4/18:0)	-0.19	1.2E-03	C00157	Lipids	Glycerophospholipids	Glycerophosphocholines	Diacylglycerophosphocholines	Down
PC(20:3/16:0)	-0.17	2.1E-03	C00157	Lipids	Glycerophospholipids	Glycerophosphocholines	Diacylglycerophosphocholines	Down
Wighteone	-0.11	2.1E-03	C10542	Lipids	Polyketides	Flavonoids	Isoflavonoids	Down
PE-Cer(d15:1(4E)/22:0(2OH))	-0.15	2.2E-03	C06062	Lipids	Sphingolipids	Phosphosphingolipids	Ceramide phosphoethanolamines	Down
Taurine**	-0.07	3.6E-03	C00245	Organic acids	none	none	none	Down
PC(20:4/18:0)	-0.12	3.8E-03	C00157	Lipids	Glycerophospholipids	Glycerophosphocholines	Diacylglycerophosphocholines	Down
PE(22:5/P-18:0)	-0.11	4.1E-03	C00350	Lipids	Glycerophospholipids	Glycerophosphoethanolamines	Diacylglycerophosphoethanolamines	Down
Piperidine	-0.06	4.1E-03	C01746	NA	NA	NA	NA	Down
PC(22:5/18:1)	-0.06	5.1E-03	C00157	Lipids	Glycerophospholipids	Glycerophosphocholines	Diacylglycerophosphocholines	Down
PE(22:4/P-18:0)	-0.11	5.1E-03	C00350	Lipids	Glycerophospholipids	Glycerophosphoethanolamines	Diacylglycerophosphoethanolamines	Down
SM(d18:0/16:1)	-0.06	6.0E-03	C00550	Lipids	Sphingolipids	Phosphosphingolipids	Ceramide phosphocholines (sphingomyelins)	Down

SM(d18:1/24:1)	-0.17	6.0E-03	C00550	Lipids	Sphingolipids	Phosphosphingolipids	Ceramide phosphocholines (sphingomyelins)	Down
Sphingosine 1-phosphate	-0.14	7.2E-03	C06124	Lipids	Sphingolipids	Sphingoid bases	Sphingoid base 1-phosphates	Down
PE(20:4/P-18:1)	-0.07	7.9E-03	C00350	Lipids	Glycerophospholipids	Glycerophosphoethano lamines	Diacylglycerophosphoethanola mines	Down
PE-Cer(d16:1(4E)/20:0)	-0.10	8.1E-03	C06062	Lipids	Sphingolipids	Phosphosphingolipids	Ceramide phosphoethanolamines	Down
PC(20:3/18:1)	-0.07	8.3E-03	C00157	Lipids	Glycerophospholipids	Glycerophosphocholin es	Diacylglycerophosphocholines	Down
SM(d18:2/20:0)	-0.15	8.8E-03	C00550	Lipids	Sphingolipids	Phosphosphingolipids	Ceramide phosphocholines (sphingomyelins)	Down
PS(22:6/21:0)	-2.43	1.1E-02	C02737	Lipids	Glycerophospholipids	Glycerophosphoserines	Diacylglycerophosphoserines	Down
PS(22:6/21:0)	-2.42	1.1E-02	C02737	Lipids	Glycerophospholipids	Glycerophosphoserines	Diacylglycerophosphoserines	Down
SM(d18:1/24:1)**	-0.18	1.1E-02	C00550	Lipids	Sphingolipids	Phosphosphingolipids	Ceramide phosphocholines (sphingomyelins)	Down
PI(20:3/18:4)	-0.08	1.2E-02	C01194	Lipids	Glycerophospholipids	Glycerophosphoinosito ls	Diacylglycerophosphoinositols	Down
PC(18:3/18:0)	-2.36	1.3E-02	C00157	Lipids	Glycerophospholipids	Glycerophosphocholin es	Diacylglycerophosphocholines	Down
SM(d18:0/16:1(OH))	-0.09	1.3E-02	C00550	Lipids	Sphingolipids	Phosphosphingolipids	Ceramide phosphocholines (sphingomyelins)	Down
Sphing-4-enine-1-phosphocholine	-0.13	1.3E-02	C03640	Lipids	Sphingolipids	Sphingoid bases	Lysosphingomyelins and lysoglycosphingolipids	Down
PC(22:4/18:1)	-0.09	1.3E-02	C00157	Lipids	Glycerophospholipids	Glycerophosphocholin es	Diacylglycerophosphocholines	Down
Delphinidin 3-(6-p-coumaroyl)glucoside	-0.07	1.5E-02	C16370	Lipids	Polyketides	Flavonoids	Anthocyanidins	Down
PS(22:6/21:0)	-2.31	1.5E-02	C02737	Lipids	Glycerophospholipids	Glycerophosphoserines	Diacylglycerophosphoserines	Down
Carnitine**	-0.05	1.6E-02	C00318	NA	NA	NA	NA	Down
SM(d18:2/24:1)**	-0.17	1.6E-02	C00550	Lipids	Sphingolipids	Phosphosphingolipids	Ceramide phosphocholines (sphingomyelins)	Down
PC(20:3/16:0)	-0.16	2.0E-02	C00157	Lipids	Glycerophospholipids	Glycerophosphocholin es	Diacylglycerophosphocholines	Down
PC(22:4/18:1)	-0.08	2.0E-02	C00157	Lipids	Glycerophospholipids	Glycerophosphocholin es	Diacylglycerophosphocholines	Down
PE(22:1/18:3)	-4.19	2.0E-02	C00350	Lipids	Glycerophospholipids	Glycerophosphoethano lamines	Diacylglycerophosphoethanola mines	Down
SM(d18:1/18:1)**	-0.12	2.0E-02	C00550	Lipids	Sphingolipids	Phosphosphingolipids	Ceramide phosphocholines (sphingomyelins)	Down

PI(18:0/20:4)	-0.06	2.1E-02	C00626	NA	NA	NA	NA	Down
PC(20:3/18:1)	-0.04	2.1E-02	C00157	Lipids	Glycerophospholipids	Glycerophosphocholin es	Diacylglycerophosphocholines	Down
PE(18:1/P-18:1)	-0.08	2.1E-02	C00350	Lipids	Glycerophospholipids	Glycerophosphoethano lamines	Diacylglycerophosphoethanola mines	Down
PE(20:3/P-18:1)	-0.04	2.1E-02	C00350	Lipids	Glycerophospholipids	Glycerophosphoethano lamines	Diacylglycerophosphoethanola mines	Down
PC(30:0)*	-0.03	2.2E-02	C00157	Lipids	Glycerophospholipids	Glycerophosphocholin es	Diacylglycerophosphocholines	Down
PC(30:0)*	-0.03	2.4E-02	C00157	Lipids	Glycerophospholipids	Glycerophosphocholin es	Di acylglycerophosphocholines	Down
PI(20:3/20:5)	-0.10	2.7E-02	C01194	Lipids	Glycerophospholipids	Glycerophosphoinosito ls	Diacylglycerophosphoinositols	Down
PC(18:0/20:5)	-0.21	2.7E-02	C00157	Lipids	Glycerophospholipids	Glycerophosphocholin es	Diacylglycerophosphocholines	Down
PC(P-14:0/18:1)	-0.06	2.7E-02	C13895	Lipids	Glycerophospholipids	Glycerophosphocholin es	1Z-alkenyl,2-acylglycerophosphocholines	Down
PS(20:4/18:0)	-0.09	2.8E-02	C02737	Lipids	Glycerophospholipids	Glycerophosphoserines	Diacylglycerophosphoserines	Down
PC(18:3/15:0)	-0.03	2.8E-02	C00157	Lipids	Glycerophospholipids	Glycerophosphocholin es	Diacylglycerophosphocholines	Down
5-Fluorodeoxyuridine	-0.06	2.9E-02	C11736	NA	NA	NA	NA	Down
PE(P-38:4)*	-0.05	2.9E-02	C00350	Lipids	Glycerophospholipids	Glycerophosphoethano lamines	Diacylglycerophosphoethanola mines	Down
PC(34:1)*	-0.03	3.0E-02	C00157	Lipids	Glycerophospholipids	Glycerophosphocholin es	Diacylglycerophosphocholines	Down
PC(18:0/18:3)	-0.04	3.4E-02	C00157	Lipids	Glycerophospholipids	Glycerophosphocholin es	Diacylglycerophosphocholines	Down
PE(18:2/P-16:0)	-0.05	3.5E-02	C00350	Lipids	Glycerophospholipids	Glycerophosphoethano lamines	Diacylglycerophosphoethanola mines	Down
PGD2	-0.09	3.6E-02	C00696	Lipids	Fatty acyls	Eicosanoids	Prostaglandins	Down
Gamma-Butyrolactone	-0.07	3.6E-02	C01770	NA	NA	NA	NA	Down
PE(18:1/18:0)	-0.17	3.9E-02	C00350	Lipids	Glycerophospholipids	Glycerophosphoethano lamines	Diacylglycerophosphoethanola mines	Down
PC(20:1/18:1)	-2.24	4.3E-02	C00157	Lipids	Glycerophospholipids	Glycerophosphocholin es	Diacylglycerophosphocholines	Down
PE(18:1/P-16:0)	-0.04	4.3E-02	C00350	Lipids	Glycerophospholipids	Glycerophosphoethano lamines	Diacylglycerophosphoethanola mines	Down
Glycerophosphocholine	-0.03	4.4E-02	C00670	NA	NA	NA	NA	Down

Cer(d18:1/24:1)	-0.06	4.5E-02	C00195	Lipids	Sphingolipids	Ceramides	N-acylsphingosines (ceramides)	Down
SM(d18:2/23:0)	-2.20	4.6E-02	C00550	Lipids	Sphingolipids	Phosphosphingolipids	Ceramide phosphocholines (sphingomyelins)	Down
SM(d18:1/24:0)	-1.62	4.7E-02	C00550	Lipids	Sphingolipids	Phosphosphingolipids	Ceramide phosphocholines (sphingomyelins)	Down
SM(d18:1/24:1)	-2.17	4.7E-02	C00550	Lipids	Sphingolipids	Phosphosphingolipids	Ceramide phosphocholines (sphingomyelins)	Down
PC(P-14:0/18:1)	-0.07	4.7E-02	C13895	Lipids	Glycerophospholipids	Glycerophosphocholin es	1Z-alkenyl,2-acylglycerophosphocholines	Down
PE(18:1/20:0)	-2.16	4.7E-02	C00350	Lipids	Glycerophospholipids	Glycerophosphoethano lamines	Diacylglycerophosphoethanola mines	Down
SM(d18:1/16:1)	-0.08	4.7E-02	C00550	Lipids	Sphingolipids	Phosphosphingolipids	Ceramide phosphocholines (sphingomyelins)	Down
Oleamide	-0.10	4.8E-02	C19670	NA	NA	NA	NA	Down
7,8-dihydro-L-Biopterin	0.12	1.9E-04	C02953	NA	NA	NA	NA	UP
Palmitoyl Ethanolamide	0.29	3.3E-04	C16512	NA	NA	NA	NA	UP
PG(16:0/0:0)	0.15	3.3E-04	C00344	NA	NA	NA	NA	UP
Sphinganine	0.27	3.3E-04	C00836	Lipids	Sphingolipids	Sphingoid bases	Sphinganines	UP
Butyryl-L-carnitine	0.11	5.1E-04	C02862	Lipids	Fatty acyls	Fatty esters	Fatty acyl carnitines	UP
2(3H)-Furanone	0.11	6.1E-04	C17602	NA	NA	NA	NA	UP
Octadecanamide	0.15	6.1E-04	C13846	Lipids	Fatty acyls	Fatty amides	Primary amides	UP
PS(18:3/17:2)	0.07	1.8E-03	C02737	Lipids	Glycerophospholipids	Glycerophosphoserines	Diacylglycerophosphoserines	UP
Hypoxanthine	0.20	2.1E-03	C00262	Phytoche mical compound s	Alkaloids	Others	Purine alkaloids	UP
N5-Dinitrophenyl-L-ornithine methyl ester	0.26	2.1E-03	C04541	NA	NA	NA	NA	UP
N2-Methylguanine	0.04	2.2E-03	C04153	NA	NA	NA	NA	UP
3,4-Dehydrothiomorpholine-3-carboxylate	0.17	2.9E-03	C04445	NA	NA	NA	NA	UP
6-Hydroxyl-1,6-dihydropurine ribonucleoside	0.24	2.9E-03	C04583	NA	NA	NA	NA	UP
LysoPC(14:0)*	0.14	2.9E-03	C04230	Lipids	Glycerophospholipids	Glycerophosphocholin es	Monoacylglycerophosphocholi nes	UP
PS(19:1/18:4)	0.12	2.9E-03	C02737	Lipids	Glycerophospholipids	Glycerophosphoserines	Diacylglycerophosphoserines	UP

LysoPC(16:1)	0.08	4.1E-03	C04230	Lipids	Glycerophospholipids	Glycerophosphocholines	Monoacylglycerophosphocholines	UP
N-Succinyl-L-diaminopimelic acid	0.17	6.1E-03	C04421	NA	NA	NA	NA	UP
Benzaldehyde	0.11	6.9E-03	C00193	NA	NA	NA	NA	UP
Avermectin A1a	0.08	7.1E-03	C11984	Lipids	Polyketides	Macrolides and lactone polyketides	none	UP
Avermectin A1a	0.08	7.6E-03	C11984	Lipids	Polyketides	Macrolides and lactone polyketides	none	UP
Macrozamin	0.10	8.3E-03	C08504	NA	NA	NA	NA	UP
5,6-Dihydroxy-3-methyl-2-oxo-1,2-dihydroquinoline	0.07	1.0E-02	C06725	NA	NA	NA	NA	UP
CYTIDINE 5′-DIPHOSPHOCHOLINE**	0.06	1.3E-02	C02354	Nucleic acids	Cyclic nucleotides	2′,3′-Cyclic nucleotides	none	UP
PS(22:6/19:0)	0.14	1.6E-02	C02737	Lipids	Glycerophospholipids	Glycerophosphoserines	Diacylglycerophosphoserines	UP
5,10-Methenyltetrahydrofolate	0.08	1.8E-02	C00445	NA	NA	NA	NA	UP
Terpendole E	0.07	1.8E-02	C20536	NA	NA	NA	NA	UP
Guanine	0.17	2.0E-02	C00242	Nucleic acids	Bases	Purines	none	UP
PS(22:6/19:1)	0.22	2.0E-02	C02737	Lipids	Glycerophospholipids	Glycerophosphoserines	Diacylglycerophosphoserines	UP
12alpha-Methylpregna-4,9(11)-diene-3,20-dione	0.20	2.0E-02	C15190	NA	NA	NA	NA	UP
Aniline	0.06	2.0E-02	C00292	NA	NA	NA	NA	UP
CL(20:1/18:2/18:2/18:1)	0.07	2.1E-02	C05980	Lipids	Glycerophospholipids	Glycerophosphoglycerophosphoglycerols	Diacylglycerophosphoglycerophosphodiradylglycerols	UP
Glaucarubin	1.58	2.1E-02	C08760	Phytochemical compounds	Terpenoids	Triterpenoids (C30)	Dammarenes	UP
Palmitoyl-L-carnitine**	0.04	2.8E-02	C02990	Lipids	Fatty acyls	Fatty esters	Fatty acyl carnitines	UP
PS(18:3/17:1)	0.07	3.4E-02	C02737	Lipids	Glycerophospholipids	Glycerophosphoserines	Diacylglycerophosphoserines	UP
PE(P-18:0/0:0)	0.06	3.4E-02	C06254	NA	NA	NA	NA	UP
PC(16:1/18:2)	0.06	3.5E-02	C00157	Lipids	Glycerophospholipids	Glycerophosphocholines	Diacylglycerophosphocholines	UP
PC(36:4)*	0.03	3.7E-02	C00157	Lipids	Glycerophospholipids	Glycerophosphocholines	Diacylglycerophosphocholines	UP

15beta-Hydroxy-7alpha-mercapto-pregn-4-ene-3,20-dione 7-acetate	0.02	3.9E-02	C14922	NA	NA	NA	NA	UP
6Œ±-methylprednisolone	0.09	4.0E-02	D00407	NA	NA	NA	NA	UP
PS(19:0/20:5)	0.15	4.0E-02	C02737	Lipids	Glycerophospholipids	Glycerophosphoserines	Diacylglycerophosphoserines	UP
PS(22:6/17:1)	0.08	4.2E-02	C02737	Lipids	Glycerophospholipids	Glycerophosphoserines	Diacylglycerophosphoserines	UP
PC(18:3/22:5)	0.04	4.3E-02	C00157	Lipids	Glycerophospholipids	Glycerophosphocholin es	Diacylglycerophosphocholines	UP
Nanchangmycin	0.22	4.3E-02	C12047	NA	NA	NA	NA	UP
PS(19:0/20:5)	0.13	4.5E-02	C02737	Lipids	Glycerophospholipids	Glycerophosphoserines	Diacylglycerophosphoserines	UP
PS(22:6/19:0)	0.14	4.5E-02	C02737	Lipids	Glycerophospholipids	Glycerophosphoserines	Diacylglycerophosphoserines	UP
PS(22:6/19:1)	0.16	4.5E-02	C02737	Lipids	Glycerophospholipids	Glycerophosphoserines	Diacylglycerophosphoserines	UP
Nanchangmycin	0.22	4.5E-02	C12047	NA	NA	NA	NA	UP
2(3H)-Furanone	0.06	4.5E-02	C17602	NA	NA	NA	NA	UP
PS(17:2/22:4)	0.16	4.7E-02	C02737	Lipids	Glycerophospholipids	Glycerophosphoserines	Diacylglycerophosphoserines	UP
Anastrozole	0.11	4.7E-02	C08159	NA	NA	NA	NA	UP
PS(22:6/19:0) :1	0.12	4.7E-02	C02737	Lipids	Glycerophospholipids	Glycerophosphoserines	Diacylglycerophosphoserines	UP
CYTOSINE**	0.05	4.7E-02	C00380	Nucleic acids	Bases	Pyrimidines	none	UP
LysoPC(16:0)*	0.03	4.8E-02	C04230	Lipids	Glycerophospholipids	Glycerophosphocholin es	Monoacylglycerophosphocholi nes	UP
PC(18:3/20:4)	0.08	4.8E-02	C00157	Lipids	Glycerophospholipids	Glycerophosphocholin es	Diacylglycerophosphocholines	UP

Table S2. Significantly up- and downregulated genes in house dust mite (HDM)-sensitized mice compared to control mice. This list includes only successfully annotated genes. Genes were ordered by p-value from small to large and by direction of fold-change.

Gene id	log2 Fold Change	P-adj	MGI symbol	Gene biotype	Function	Go id	Regulation
ENSMUSG00000055730	-1.2364234	5.09E-23	Ces2a	protein_coding	retinol metabolic process	GO:0042572	Down
ENSMUSG00000011751	-1.7130549	7.83E-22	Sptbn4	protein_coding	transmission of nerve impulse	GO:0019226	Down
ENSMUSG00000019301	-0.461028	7.49E-05	Hsd17b1	protein_coding	cellular response to metal ion	GO:0071248	Down
ENSMUSG00000033768	-0.9080318	7.50E-05	Nrxn2	protein_coding	protein binding	GO:0005515	Down
ENSMUSG00000049092	-0.4733845	2.24E-04	Gpr137c	protein_coding			Down
ENSMUSG00000033634	-0.6646306	3.83E-04	Nat8f2	protein_coding	integral component of membrane	GO:0016021	Down
ENSMUSG00000118257	-0.6526469	3.83E-04	9630014M24Rik	lncRNA			Down
ENSMUSG00000081819	-0.6327779	4.01E-04	Gm12722	processed_pseudogene			Down
ENSMUSG00000100068	-1.2361447	4.35E-04	Gm18558	processed_pseudogene			Down
ENSMUSG00000035778	-0.4690194	6.35E-04	Ggta1	protein_coding	membrane	GO:0016020	Down
ENSMUSG00000110290	-0.5912128	6.65E-04	Gm45336	lncRNA			Down
ENSMUSG00002076269	-0.9945969	6.65E-04	7SK	misc_RNA			Down
ENSMUSG00000004056	-0.5318352	7.77E-04	Akt2	protein_coding	metal ion binding	GO:0046872	Down
ENSMUSG00000036646	-0.5418483	7.77E-04	Man1b1	protein_coding	hydrolase activity, acting on glycosyl bonds	GO:0016798	Down
ENSMUSG00000042359	-1.3003126	0.00115698	Osbpl6	protein_coding	perinuclear endoplasmic reticulum	GO:0097038	Down
ENSMUSG00000103357	-1.0584714	0.00186319	Gm7804	transcribed_processed_pseudogene			Down
ENSMUSG00000052751	-0.5827251	0.00207556	Repin1	protein_coding	chromatin insulator sequence binding	GO:0043035	Down
ENSMUSG00000116506	-1.4686672	0.00236419	5730414N17Rik	lncRNA			Down
ENSMUSG00000062169	-0.5441042	0.00297261	Cnih4	protein_coding	vesicle-mediated transport	GO:0016192	Down
ENSMUSG00000058435	-0.6742729	0.00475252	Btnl4	protein_coding	integral component of membrane	GO:0016021	Down
ENSMUSG00000027628	-0.384203	0.00483484	Aar2	protein_coding	cellular_component	GO:0005575	Down
ENSMUSG00000031824	-1.3955174	0.00653992	6430548M08Rik	protein_coding	molecular_function	GO:0003674	Down

ENSMUSG00000117011	-0.4440847	0.00675888	E130008D07Rik	lncRNA			Down
ENSMUSG00000054263	-0.4946908	0.00713124	Lifr	protein_coding	cytokine-mediated signaling pathway	GO:0019221	Down
ENSMUSG00000022754	-1.1712	0.00764356	Tmem45a	protein_coding			Down
ENSMUSG00000024603	-0.4008169	0.00834816	Dctn4	protein_coding	centrosome	GO:0005813	Down
ENSMUSG00000034218	-0.4275452	0.00834816	Atm	protein_coding	telomere maintenance	GO:0000723	Down
ENSMUSG00000026721	-0.3630345	0.00972313	Rabgap1l	protein_coding	protein transport	GO:0015031	Down
ENSMUSG00000027950	-1.1134603	0.00972313	Chrnb2	protein_coding	regulation of circadian sleep/wake cycle, REM sleep	GO:0042320	Down
ENSMUSG00000028710	-0.8637415	0.01035835	Atpaf1	protein_coding	molecular_function	GO:0003674	Down
ENSMUSG00000021621	-1.207908	0.01119512	Zcchc9	protein_coding			Down
ENSMUSG00000024999	-0.9734825	0.01440233	Noc3l	protein_coding	mitochondrion	GO:0005739	Down
ENSMUSG00000041133	-0.7488429	0.01488775	Smc1a	protein_coding	cellular response to DNA damage stimulus	GO:0006974	Down
ENSMUSG00000071073	-0.4626434	0.01633402	Lrrc73	protein_coding	molecular_function	GO:0003674	Down
ENSMUSG00000032921	-0.8695015	0.01779115	Odf4	protein_coding	motile cilium	GO:0031514	Down
ENSMUSG00000043091	-0.4999434	0.0183228	Tuba1c	protein_coding	microtubule-based process	GO:0007017	Down
ENSMUSG00000030120	-0.5449588	0.02115965	Mlf2	protein_coding			Down
ENSMUSG00000050271	-0.3266236	0.02382354	Prag1	protein_coding	cytoplasm	GO:0005737	Down
ENSMUSG00000025239	-0.4285294	0.02754945	Limd1	protein_coding	osteoblast development	GO:0002076	Down
ENSMUSG00000031337	-0.3962409	0.02754945	Mtm1	protein_coding	lipid metabolic process	GO:0006629	Down
ENSMUSG00000074776	-1.2281625	0.02754945	Gm10754	lncRNA			Down
ENSMUSG00000110666	-0.4275773	0.02754945	Gm9172	processed_pseudogene			Down
ENSMUSG00002075875	-0.5519522	0.02775445	SNORA62	snoRNA			Down
ENSMUSG00000058655	-0.4127737	0.02915549	Eif4b	protein_coding	postsynapse	GO:0098794	Down
ENSMUSG00000002319	-0.5647162	0.03008777	Ipo4	protein_coding	ribosome biogenesis	GO:0042254	Down
ENSMUSG00000032064	-0.6245032	0.0349174	Dixdc1	protein_coding	cerebral cortex radially oriented cell migration	GO:0021799	Down
ENSMUSG00000026274	-0.6357913	0.03737922	Pask	protein_coding	ATP binding	GO:0005524	Down
ENSMUSG00000023044	-0.2576511	0.04203729	Csad	protein_coding	sulfinoalanine decarboxylase activity	GO:0004782	Down

ENSMUSG00000043144	-1.615987	0.04347489	Aqp6	protein_coding	odontogenesis	GO:0042476	Down
ENSMUSG00000096553	-0.3453126	0.04408523	Gm10097	lncRNA			Down
ENSMUSG00000026478	-0.7761952	0.0456216	Lamc1	protein_coding	hemidesmosome assembly	GO:0031581	Down
ENSMUSG00000056305	-0.3798892	0.0456216	Usp39	protein_coding	cell division	GO:0051301	Down
ENSMUSG00000085041	-0.8364465	0.04763079	BB031773	lncRNA	molecular_function	GO:0003674	Down
ENSMUSG00000071036	-0.3358023	0.04873516	Gm10309	protein_coding	molecular_function	GO:0003674	Down
ENSMUSG00000085196	-0.4577486	0.04873516	Gm14963	lncRNA			Down
ENSMUSG00000028197	10.531171	5.09E-23	Col24a1	protein_coding	collagen-containing extracellular matrix	GO:0062023	Up
ENSMUSG00000079741	2.68914709	1.20E-19	Nlrp4g	transcribed_unprocessed_pseudogene			Up
ENSMUSG00000109336	3.99222285	1.61E-12	Samd4b	protein_coding	cytoplasm	GO:0005737	Up
ENSMUSG00000003444	4.31663869	7.95E-12	Med29	protein_coding	mediator complex	GO:0016592	Up
ENSMUSG00000106373	7.00009108	1.13E-07	Gm6522	unprocessed_pseudogene			Up
ENSMUSG00000071650	1.09532617	3.93E-07	Ganab	protein_coding	carbohydrate metabolic process	GO:0005975	Up
ENSMUSG00000020255	0.67054155	5.60E-07	Nopchap1	protein_coding	nucleus	GO:0005634	Up
ENSMUSG00000064337	2.02279914	1.35E-06	mt-Rnr1	Mt_rRNA	ribosomal small subunit assembly	GO:0000028	Up
ENSMUSG00000106248	4.46816804	2.67E-06	Gm9728	processed_pseudogene			Up
ENSMUSG00000046808	3.07392162	3.49E-06	Atp10d	polymorphic_pseudogene	integral component of membrane	GO:0016021	Up
ENSMUSG00000079101	4.23898551	1.18E-05	Esd-ps	processed_pseudogene			Up
ENSMUSG00000099851	3.8850197	1.18E-05	Gm29542	lncRNA			Up
ENSMUSG00000063779	2.01966728	1.33E-05	Chil4	protein_coding	inflammatory response	GO:0006954	Up
ENSMUSG00000037474	2.20928324	1.82E-05	Dtl	protein_coding	regulation of cell cycle	GO:0051726	Up
ENSMUSG00000024330	0.85460194	2.24E-05	Col11a2	protein_coding	metal ion binding	GO:0046872	Up
ENSMUSG00000028879	1.52715214	1.17E-04	Stx12	protein_coding	protein binding	GO:0005515	Up
ENSMUSG00000019905	1.17834913	1.82E-04	Gprc6a	protein_coding	membrane	GO:0016020	Up
ENSMUSG00000020156	0.55847617	2.34E-04	Pwwp3a	protein_coding	nucleoplasm	GO:0005654	Up
ENSMUSG00000096345	5.38701683	2.39E-04	Esp16	protein_coding	biological_process	GO:0008150	Up

ENSMUSG00000027902	5.70752212	2.53E-04	Chil6	protein_coding	positive regulation of chemokine production	GO:0032722	Up
ENSMUSG00000040829	2.5799862	4.01E-04	Zmynd15	protein_coding	nucleus	GO:0005634	Up
ENSMUSG00000064339	1.92660563	6.35E-04	mt-Rnr2	Mt_rRNA	translation	GO:0006412	Up
ENSMUSG00000104211	4.65831304	6.35E-04	Gm37985	TEC			Up
ENSMUSG00000108290	1.66993305	6.64E-04	Gm44120	TEC			Up
ENSMUSG00000005803	1.80566343	7.07E-04	Sqor	protein_coding	quinone binding	GO:0048038	Up
ENSMUSG00000021922	0.68396419	7.07E-04	Itih4	protein_coding	acute-phase response	GO:0006953	Up
ENSMUSG00000048521	2.31995382	0.00115698	Cxcr6	protein_coding	immune response	GO:0006955	Up
ENSMUSG00000000248	1.33805444	0.00204658	Clec2g	protein_coding			Up
ENSMUSG00000020014	5.50847975	0.00234764	Cfap54	protein_coding	cell projection organization	GO:0030030	Up
ENSMUSG00000071793	5.50544873	0.00267493	2610005L07Rik	transcribed_unprocessed_pseudogene			Up
ENSMUSG00000090455	3.22172954	0.00273636	Gm2431	unprocessed_pseudogene			Up
ENSMUSG00000001225	1.23313621	0.00333248	Slc26a3	protein_coding	integral component of membrane	GO:0016021	Up
ENSMUSG00000034781	0.66041725	0.00368955	Gna11	protein_coding			Up
ENSMUSG00000006526	0.70633887	0.00374412	Stimate	protein_coding	integral component of membrane	GO:0016021	Up
ENSMUSG00000090726	5.11599889	0.00483484	Gm17421	unprocessed_pseudogene			Up
ENSMUSG00000021313	4.15524447	0.00488022	Ryr2	protein_coding	negative regulation of cytosolic calcium ion concentration	GO:0051481	Up
ENSMUSG00000026141	1.13842436	0.00507061	Col19a1	protein_coding	extracellular matrix organization	GO:0030198	Up
ENSMUSG00000034751	5.14939466	0.00522295	Mast4	protein_coding	protein serine/threonine kinase activity	GO:0004674	Up
ENSMUSG00000064357	0.9987857	0.00522295	mt-Atp6	protein_coding	ATP synthesis coupled proton transport	GO:0015986	Up
ENSMUSG00000115801	1.91422642	0.00543413		lncRNA	gene silencing by miRNA	GO:0035195	Up
ENSMUSG00000095891	5.33056591	0.00562315	Gm10717	protein_coding	cellular_component	GO:0005575	Up
ENSMUSG00000058006	4.65246182	0.00735682	Mdn1	protein_coding			Up
ENSMUSG00000101609	4.75207683	0.00972313	Kcnq1ot1	lncRNA	nucleolus	GO:0005730	Up
ENSMUSG00000118666	5.28927852	0.00972313	Gm46426	Processed pseudogene			Up

ENSMUSG00000111194	1.60164078	0.01054145	Gm47180	Transcribed unprocessed pseudogene			Up
ENSMUSG00000098197	0.63610378	0.01114418	BC051537	lncRNA			Up
ENSMUSG00000015357	2.04088186	0.01119512	Clpx	protein_coding	nucleotide binding	GO:0000166	Up
ENSMUSG00000016942	0.94995088	0.01169093	Tmprss6	protein_coding	proteolysis	GO:0006508	Up
ENSMUSG00000094098	5.33546486	0.01202867	Vmn2r44	protein_coding	signal transduction	GO:0007165	Up
ENSMUSG00000005268	4.97882906	0.01440233	Prlr	protein_coding	activation of Janus kinase activity	GO:0042976	Up
ENSMUSG00000028456	4.74521457	0.01488775	Unc13b	protein_coding	membrane	GO:0016020	Up
ENSMUSG00000021192	1.38643476	0.01633402	Golga5	protein_coding	Golgi vesicle transport	GO:0048193	Up
ENSMUSG00000022220	4.9755876	0.01633402	Adcy4	protein_coding	membrane	GO:0016020	Up
ENSMUSG00000075249	4.79664975	0.01633402	Fsip2	protein_coding	sperm principal piece	GO:0097228	Up
ENSMUSG00000091455	5.00006114	0.01741593	Otogl	protein_coding	extracellular matrix	GO:0031012	Up
ENSMUSG00000029282	4.16182834	0.01779115	Amtn	protein_coding	biomineral tissue development	GO:0031214	Up
ENSMUSG00000109446	4.94856286	0.01913634	Gm9195	protein_coding	molecular_function	GO:0003674	Up
ENSMUSG00000012819	0.62771784	0.02100476	Cdh23	protein_coding	synapse	GO:0045202	Up
ENSMUSG00000049252	4.86127615	0.02154792	Lrp1b	protein_coding	endocytosis	GO:0006897	Up
ENSMUSG00000059762	0.4988544	0.022738	Olfr767	protein_coding	olfactory receptor activity	GO:0004984	Up
ENSMUSG00000056211	1.5591643	0.0229522	R3hdm1	protein_coding	biological_process	GO:0008150	Up
ENSMUSG00000021318	2.95548746	0.02375362	Gli3	protein_coding	nucleoplasm	GO:0005654	Up
ENSMUSG00000027332	0.85114704	0.026354	Ivd	protein_coding	branched-chain amino acid catabolic process	GO:0009083	Up
ENSMUSG00000056602	4.99715516	0.02681502	Fry	protein_coding			Up
ENSMUSG00000023032	1.08769523	0.02754945	Slc4a8	protein_coding	symmetric synapse	GO:0032280	Up
ENSMUSG00000059921	4.67528406	0.02754945	Unc5c	protein_coding	lamellipodium	GO:0030027	Up
ENSMUSG00000037270	4.58000717	0.02967399	4932438A13Rik	protein_coding	fat cell differentiation	GO:0045444	Up
ENSMUSG00000044768	4.50536302	0.03234886	Macir	protein_coding	negative regulation of cytokine production involved in inflammatory response	GO:1900016	Up
ENSMUSG00000025347	1.18042368	0.03608577	Mettl7b	protein_coding	methyltransferase activity	GO:0008168	Up

ENSMUSG00000025907	4.72061611	0.03737922	Rb1cc1	protein_coding	phagophore assembly site membrane	GO:0034045	Up
ENSMUSG00000096141	4.93760864	0.03737922	Dnah7a	protein_coding	calcium ion binding	GO:0005509	Up
ENSMUSG00000036990	4.25777368	0.03884582	Otud4	protein_coding	nucleus	GO:0005634	Up
ENSMUSG00000091491	5.10264639	0.04111617	Vmn2r97	protein_coding	signal transduction	GO:0007165	Up
ENSMUSG00000087384	1.06443939	0.04203729	Gm15558	lncRNA	cellular_component	GO:0005575	Up
ENSMUSG00000051329	0.39272824	0.04335361	Nup160	protein_coding	kinetochore	GO:0000776	Up
ENSMUSG00000061601	4.32872756	0.04335361	Pclo	protein_coding	transport vesicle	GO:0030133	Up
ENSMUSG00000034156	4.93327139	0.04436161	Tspoap1	protein_coding	calyx of Held	GO:0044305	Up
ENSMUSG00000040118	4.79212325	0.0444634	Cacna2d1	protein_coding	voltage-gated calcium channel complex	GO:0005891	Up
ENSMUSG00000004668	4.96628015	0.0456216	Abca13	protein_coding			Up
ENSMUSG00000029370	2.30393492	0.0456216	Rassf6	protein_coding	cellular_component	GO:0005575	Up
ENSMUSG00000053819	5.06547329	0.0456216	Camk2d	protein_coding	kinase activity	GO:0016301	Up
ENSMUSG00000055204	4.16816638	0.0456216	Ankrd17	protein_coding	defense response to bacterium	GO:0042742	Up
ENSMUSG00000025326	4.92130561	0.04573903	Ube3a	protein_coding	ubiquitin-dependent protein catabolic process	GO:0006511	Up
ENSMUSG00000096373	4.57184579	0.04763079	Vmn2r31	protein_coding	integral component of plasma membrane	GO:0005887	Up
ENSMUSG00000075376	4.529686	0.04801771	Rc3h2	protein_coding	spleen development	GO:0048536	Up
ENSMUSG00000035954	4.20172352	0.04873516	Dock4	protein_coding	cytoplasm	GO:0005737	Up
ENSMUSG00000040928	0.57122702	0.04873516	S100pbp	protein_coding	nuclear speck	GO:0016607	Up
ENSMUSG00000024335	2.09922702	0.04928528	Brd2	protein_coding	nucleosome assembly	GO:0006334	Up

Table S3. Relevant genes' function literature review. A literature review was conducted to determine potential relevance of significant genes to allergy and/or asthma.

	Gene name	Functions	Literature support
Top five upregulated genes	Col24a	Immune	Participate in regulating type I collagen fibrillogenesis [3] and differentially expressed in T-cell of obese asthmatics compared to normal weight asthmatics [4]
	Nlrp4g	Immune	Plays a pivotal role in the innate immune and reproductive systems in the mouse and human [5-9]
	Samd4b		Overexpression inhibits the transcriptional activities of AP-1, p53/TP53 and CDKN1A[10] and nasal tissue expression increased with severity of hyperinflation in COPD patients [11]
	Med29		A general transcription factor of RNA polymerase II [12] and implicated in tumor suppression effect through control of cell cycle and cell division [13]
	Ganab	Immune	Encodes the alpha subunit of glucosidase II and a member of the glycosyl hydrolase 31 family of proteins which are involved in Glycosylation of proteins [14] and lipids (glycosphingolipids) [15] and plays a role in the maturation process of both innate and adaptive immune responses [16,17]
Upregulated immune genes	Chil4	Immune	Enables chitin binding activity and kinase binding activity, has low chemotactic activity for eosinophils, and may play a role in inflammation and allergy [18,19]
	Chil6	Immune	Degrades chitin and chitotriose, plays a role in T-helper cell type 2 (Th2) immune response (contributes to the response to IL-13 and inflammation in response to IL-13), stimulates chemokine production by pulmonary epithelial cells, and protects lung epithelial cells against apoptosis and promotes phosphorylation of AKT1 [20-23] and associated with asthma [24,25]
	Cxcr6	Immune	A chemokine receptor with unique binding to CXCL16, which can exist in transmembrane and soluble forms and produced by epithelial and immune cells and can serve as an alarmin to recruit cells to the site of inflammation[26-28]
	Macir	Immune	A regulator of macrophage function, by inhibiting glycolysis and enhancing the resolution of inflammation and wound repair functions mediated by M2 macrophages [29]

	Clec2g	Immune	Encodes a member of the natural killer cell receptor C-type lectin family is a receptor for KLRB1 that protects target cells against natural killer cell-mediated lysis [30], inhibits osteoclast formation [31], and modulates the release of interferon-gamma [32]
Upregulated metabolism genes	Atp10d	Metabolism	A lipid metabolism related upregulated gene that enables glycosylceramide flippase activity and predicted to be involved in phospholipid translocation [33]
	Ivd	Metabolism	Catalyzes the conversion of isovaleryl-CoA/3-methylbutanoyl-CoA to 3-methylbut-2-enoyl-CoA as an intermediate step in the leucine (Leu) catabolic pathway and catalyzes the oxidation of other saturated short-chain acyl-CoA thioesters as pentanoyl-CoA, hexenoyl-CoA and butenoyl-CoA [34]
Top downregulated genes	Ces2a	Metabolism	Hydrolyze a wide range of xenobiotic and endogenous compounds, including lipid esters such as triacylglycerol (TG), diacylglycerol (DG), and endocannabinoid 2-arachidonoylglycerol (2-AG) and its cyclooxygenase (COX)-derived prostaglandin glyceryl esters [35-38]
	Sptbn4	Metabolism	Variants of this gene disrupt the cytoskeletal machinery that controls proper localization of ion channels and the function of axonal domains, thereby generating severe neurological dysfunction [39,40]
	Hsd17b1	Metabolism	Belongs to the short-chain dehydrogenases/reductases (17 beta-Hydroxysteroid dehydrogenases/17-ketosteroid reductases, 17HSDs) which modulates the biological activity of certain estrogens and androgens by catalyzing reductase or dehydrogenase reactions between 17-keto- and 17 beta-hydroxysteroids [41]
	Nrxn2		Encodes a member of the neurexin gene family protein products that function as cell adhesion molecules and receptors in the vertebrate nervous system [42,43]
	Gpr137c	Immune	May be involved in positive regulation of TORC1 signaling [44]
Downregulated immune genes	Btnl4		Expressed specifically by thymic epithelial cells and keratinocytes involved in T cell receptor signaling pathway and regulation of cytokine production [45,46]

	Lifr	Immune	Encodes a protein that belongs to the type I cytokine receptor family (a subunit of a receptor for leukemia inhibitory factor) [47,48]
	Atm	Immune	plays a role in pre-B cell allelic exclusion, a process leading to expression of a single immunoglobulin heavy chain allele to enforce clonality and monospecific recognition by the B-cell antigen receptor (BCR) expressed on individual B-lymphocytes [49]
	Zcchc9	Immune	Downregulates transcription mediated by NF-kappa-B [50]
Downregulated metabolism genes	Mtm1	Metabolism	Encodes a dual-specificity phosphatase that acts on both phosphotyrosine (dephosphorylate phosphotyrosine- and phosphoserine-containing peptides)[51] and phosphoserine (dephosphorylates phosphatidylinositol 3-monophosphate (PI3P) and phosphatidylinositol 3,5-bisphosphate [PI(3,5)P2]) [52-55]
	Ggta	Metabolism	Synthesizes the galactose-alpha(1,3)-galactose group by catalyzing the transfer of a galactose residue, with an alpha-1,3 linkage, on terminal lactosaminide (Gal-beta-1,4-GlcNAc-R) disaccharide borne by a glycoprotein or a glycolipid [56]
	Akt2	Metabolism	A serine/threonine kinase that is an effector molecule in the insulin signaling pathway linked to insulin's metabolic effects and lipodystrophies [57]
			REPIN1 which plays a role in glucose and lipid metabolism by differentially affecting the expression of REPIN1 target genes including glucose and fatty acid transporters [58]
	RabGAP1L	Metabolism	Recruited by ANK2 to phosphatidylinositol 3-phosphate (PI3P)-positive early endosomes, where it inactivates RAB22A, and promotes polarized trafficking to the leading edge of the migrating cells [59]
	Pask	Metabolism	Involved in energy homeostasis and protein translation under changing environmental conditions (oxygen, glucose, and nutrition) [60] and participate in respiratory regulation [61]
	Csad	Metabolism	Catalyzes the decarboxylation of L-aspartate, 3-sulfino-L-alanine (cysteine sulfinic acid), and L-cysteate to beta-alanine, hypotaurine and taurine, respectively [62,63]

Supplement references

1. Depner, C.M.; Cogswell, D.T.; Bisesi, P.J.; Markwald, R.R.; Cruickshank-Quinn, C.; Quinn, K.; Melanson, E.L.; Reisdorph, N.; Wright, K.P., Jr. Developing preliminary blood metabolomics-based biomarkers of insufficient sleep in humans. *Sleep* **2020**, *43*, doi:10.1093/sleep/zsz321.
2. Quinn, K.D.; Schedel, M.; Nkrumah-Elie, Y.; Joetham, A.; Armstrong, M.; Cruickshank-Quinn, C.; Reisdorph, R.; Gelfand, E.W.; Reisdorph, N. Dysregulation of metabolic pathways in a mouse model of allergic asthma. *Allergy* **2017**, *72*, 1327-1337, doi:https://doi.org/10.1111/all.13144.
3. Koch, M.; Laub, F.; Zhou, P.; Hahn, R.A.; Tanaka, S.; Burgeson, R.E.; Gerecke, D.R.; Ramirez, F.; Gordon, M.K. Collagen XXIV, a vertebrate fibrillar collagen with structural features of invertebrate collagens: selective expression in developing cornea and bone. *J Biol Chem* **2003**, *278*, 43236-43244, doi:10.1074/jbc.M302112200.
4. Rastogi, D.; Nico, J.; Johnston, A.D.; Tobias, T.A.M.; Jorge, Y.; Macian, F.; Greally, J.M. CDC42-related genes are upregulated in helper T cells from obese asthmatic children. *J Allergy Clin Immunol* **2018**, *141*, 539-548 e537, doi:10.1016/j.jaci.2017.04.016.
5. Peng, H.; Zhang, W.; Xiao, T.; Zhang, Y. <i>Nlrp4g</i> is an oocyte-specific gene but is not required for oocyte maturation in the mouse. *Reproduction, Fertility and Development* **2014**, *26*, 758-768, doi:https://doi.org/10.1071/RD12409.
6. An, T.; Li, S.; Pan, W.; Tien, P.; Zhong, B.; Shu, H.-B.; Wu, S. DYRK2 negatively regulates type I interferon induction by promoting TBK1 degradation via Ser527 phosphorylation. *PLoS Pathogens* **2015**, *11*, e1005179.
7. Cui, J.; Li, Y.; Zhu, L.; Liu, D.; Songyang, Z.; Wang, H.Y.; Wang, R.-F. NLRP4 negatively regulates type I interferon signaling by targeting the kinase TBK1 for degradation via the ubiquitin ligase DTX4. *Nature immunology* **2012**, *13*, 387-395.
8. Lian, Y.-G.; Zhao, H.-Y.; Wang, S.-J.; Xu, Q.-L.; Xia, X.-J. NLRP4 is an essential negative regulator of fructose-induced cardiac injury in vitro and in vivo. *Biomedicine & Pharmacotherapy* **2017**, *91*, 590-601.
9. Lin, M.; Zhao, Z.; Yang, Z.; Meng, Q.; Tan, P.; Xie, W.; Qin, Y.; Wang, R.-F.; Cui, J. USP38 inhibits type I interferon signaling by editing TBK1 ubiquitination through NLRP4 signalosome. *Molecular cell* **2016**, *64*, 267-281.
10. Luo, N.; Li, G.; Li, Y.; Fan, X.; Wang, Y.; Ye, X.; Mo, X.; Zhou, J.; Yuan, W.; Tan, M.; et al. SAMD4B, a novel SAM-containing protein, inhibits AP-1-, p53- and p21-mediated transcriptional activity. *BMB Rep* **2010**, *43*, 355-361, doi:10.5483/bmbrep.2010.43.5.355.

11. Faiz, A.; Imkamp, K.; van der Wiel, E.; Boudewijn, I.M.; Koppelman, G.H.; Brandsma, C.A.; Kerstjens, H.A.M.; Timens, W.; Vroegop, S.; Pasma, H.R.; et al. Identifying a nasal gene expression signature associated with hyperinflation and treatment response in severe COPD. *Sci Rep* **2020**, *10*, 17415, doi:10.1038/s41598-020-72551-0.
12. Mittler, G.; Kremmer, E.; Timmers, H.T.; Meisterernst, M. Novel critical role of a human Mediator complex for basal RNA polymerase II transcription. *EMBO Rep* **2001**, *2*, 808-813, doi:10.1093/embo-reports/kve186.
13. Kuuselo, R.; Savinainen, K.; Sandström, S.; Autio, R.; Kallioniemi, A. MED29, a component of the mediator complex, possesses both oncogenic and tumor suppressive characteristics in pancreatic cancer. *International journal of cancer* **2011**, *129*, 2553-2565.
14. Spiro, R.G. Protein glycosylation: nature, distribution, enzymatic formation, and disease implications of glycopeptide bonds. *Glycobiology* **2002**, *12*, 43R-56R, doi:10.1093/glycob/12.4.43r.
15. Gagneux, P.; Varki, A. Evolutionary considerations in relating oligosaccharide diversity to biological function. *Glycobiology* **1999**, *9*, 747-755, doi:10.1093/glycob/9.8.747.
16. Rudd, P.M.; Elliott, T.; Cresswell, P.; Wilson, I.A.; Dwek, R.A. Glycosylation and the immune system. *Science* **2001**, *291*, 2370-2376, doi:10.1126/science.291.5512.2370.
17. Zabczynska, M.; Pochec, E. [The role of protein glycosylation in immune system]. *Postepy Biochem* **2015**, *61*, 129-137.
18. Webb, D.C.; McKenzie, A.N.; Foster, P.S. Expression of the Ym2 lectin-binding protein is dependent on interleukin (IL)-4 and IL-13 signal transduction: identification of a novel allergy-associated protein. *J Biol Chem* **2001**, *276*, 41969-41976, doi:10.1074/jbc.M106223200.
19. Wang, L.; Ren, W.; Li, X.; Zhang, X.; Tian, H.; Bhattarai, J.P.; Challis, R.C.; Lee, A.C.; Zhao, S.; Yu, H.; et al. Chitinase-Like Protein Ym2 (Chil4) Regulates Regeneration of the Olfactory Epithelium via Interaction with Inflammation. *J Neurosci* **2021**, *41*, 5620-5637, doi:10.1523/jneurosci.1601-20.2021.
20. Seibold, M.A.; Reese, T.A.; Choudhry, S.; Salam, M.T.; Beckman, K.; Eng, C.; Atakilit, A.; Meade, K.; Lenoir, M.; Watson, H.G.; et al. Differential enzymatic activity of common haplotypic versions of the human acidic Mammalian chitinase protein. *J Biol Chem* **2009**, *284*, 19650-19658, doi:10.1074/jbc.M109.012443.
21. Boot, R.G.; Blommaart, E.F.; Swart, E.; Ghauharali-van der Vlugt, K.; Bijl, N.; Moe, C.; Place, A.; Aerts, J.M. Identification of a novel acidic mammalian chitinase distinct from chitotriosidase. *J Biol Chem* **2001**, *276*, 6770-6778, doi:10.1074/jbc.M009886200.

22. Hartl, D.; He, C.H.; Koller, B.; Da Silva, C.A.; Homer, R.; Lee, C.G.; Elias, J.A. Acidic mammalian chitinase is secreted via an ADAM17/epidermal growth factor receptor-dependent pathway and stimulates chemokine production by pulmonary epithelial cells. *J Biol Chem* **2008**, *283*, 33472-33482, doi:10.1074/jbc.M805574200.
23. Hartl, D.; He, C.H.; Koller, B.; Da Silva, C.A.; Kobayashi, Y.; Lee, C.G.; Flavell, R.A.; Elias, J.A. Acidic mammalian chitinase regulates epithelial cell apoptosis via a chitinolytic-independent mechanism. *J Immunol* **2009**, *182*, 5098-5106, doi:10.4049/jimmunol.0803446.
24. Sadeghi, H.R.; Pirayesh, A.; Shahsavan, S.; Amani, S.; Amini, S.A.; Samani, K.G.; Deris, F. Correlation of acidic mammalian chitinase expression with disease severity in patients with moderate/severe persistent allergic rhinitis. *Cent Eur J Immunol* **2020**, *45*, 294-300, doi:10.5114/ceji.2020.101251.
25. Weber-Chrysochoou, C.; Darcan-Nicolaisen, Y.; Wohlgensinger, J.; Tinner, E.M.; Frei, R.; Loeliger, S.; Lauener, R.P.; Hamelmann, E. Chitinase-Induced Airway Hyperreactivity and Inflammation in a Mouse Model of Nonallergic Asthma. *Int Arch Allergy Immunol* **2021**, *182*, 563-570, doi:10.1159/000513296.
26. Matloubian, M.; David, A.; Engel, S.; Ryan, J.E.; Cyster, J.G. A transmembrane CXC chemokine is a ligand for HIV-coreceptor Bonzo. *Nat Immunol* **2000**, *1*, 298-304, doi:10.1038/79738.
27. Day, C.; Patel, R.; Guillen, C.; Wardlaw, A.J. The chemokine CXCL16 is highly and constitutively expressed by human bronchial epithelial cells. *Exp Lung Res* **2009**, *35*, 272-283, doi:10.1080/01902140802635517.
28. Tabata, S.; Kadowaki, N.; Kitawaki, T.; Shimaoka, T.; Yonehara, S.; Yoshie, O.; Uchiyama, T. Distribution and kinetics of SR-PSOX/CXCL16 and CXCR6 expression on human dendritic cell subsets and CD4+ T cells. *J Leukoc Biol* **2005**, *77*, 777-786, doi:10.1189/jlb.1204733.
29. Dorris, E.R.; Tazzyman, S.J.; Moylett, J.; Ramamoorthi, N.; Hackney, J.; Townsend, M.; Muthana, M.; Lewis, M.J.; Pitzalis, C.; Wilson, A.G. The Autoimmune Susceptibility Gene C5orf30 Regulates Macrophage-Mediated Resolution of Inflammation. *J Immunol* **2019**, *202*, 1069-1078, doi:10.4049/jimmunol.1801155.
30. Germain, C.; Bihl, F.; Zahn, S.; Poupon, G.; Dumaurier, M.J.; Rampanarivo, H.H.; Padkjær, S.B.; Spee, P.; Braud, V.M. Characterization of alternatively spliced transcript variants of CLEC2D gene. *J Biol Chem* **2010**, *285*, 36207-36215, doi:10.1074/jbc.M110.179622.
31. Gange, C.T.; Quinn, J.M.; Zhou, H.; Kartsogiannis, V.; Gillespie, M.T.; Ng, K.W. Characterization of sugar binding by osteoclast inhibitory lectin. *J Biol Chem* **2004**, *279*, 29043-29049, doi:10.1074/jbc.M312518200.

32. Mathew, P.A.; Chuang, S.S.; Vaidya, S.V.; Kumaresan, P.R.; Boles, K.S.; Pham, H.T. The LLT1 receptor induces IFN-gamma production by human natural killer cells. *Mol Immunol* **2004**, *40*, 1157-1163, doi:10.1016/j.molimm.2003.11.024.
33. Flamant, S.; Pescher, P.; Lemercier, B.; Clément-Ziza, M.; Képès, F.; Fellous, M.; Milon, G.; Marchal, G.; Besmond, C. Characterization of a putative type IV aminophospholipid transporter P-type ATPase. *Mamm Genome* **2003**, *14*, 21-30, doi:10.1007/s00335-002-3032-3.
34. Park, J.; Chen, Y.; Tishkoff, D.X.; Peng, C.; Tan, M.; Dai, L.; Xie, Z.; Zhang, Y.; Zwaans, B.M.; Skinner, M.E.; et al. SIRT5-mediated lysine desuccinylation impacts diverse metabolic pathways. *Mol Cell* **2013**, *50*, 919-930, doi:10.1016/j.molcel.2013.06.001.
35. Alam, M.; Ho, S.; Vance, D.E.; Lehner, R. Heterologous expression, purification, and characterization of human triacylglycerol hydrolase. *Protein Expr Purif* **2002**, *24*, 33-42, doi:10.1006/prep.2001.1553.
36. Ruby, M.A.; Massart, J.; Hunerdosse, D.M.; Schönke, M.; Correia, J.C.; Louie, S.M.; Ruas, J.L.; Näslund, E.; Nomura, D.K.; Zierath, J.R. Human Carboxylesterase 2 Reverses Obesity-Induced Diacylglycerol Accumulation and Glucose Intolerance. *Cell Rep* **2017**, *18*, 636-646, doi:10.1016/j.celrep.2016.12.070.
37. Xie, S.; Borazjani, A.; Hatfield, M.J.; Edwards, C.C.; Potter, P.M.; Ross, M.K. Inactivation of lipid glyceryl ester metabolism in human THP1 monocytes/macrophages by activated organophosphorus insecticides: role of carboxylesterases 1 and 2. *Chem Res Toxicol* **2010**, *23*, 1890-1904, doi:10.1021/tx1002194.
38. Wang, R.; Borazjani, A.; Matthews, A.T.; Mangum, L.C.; Edelmann, M.J.; Ross, M.K. Identification of palmitoyl protein thioesterase 1 in human THP1 monocytes and macrophages and characterization of unique biochemical activities for this enzyme. *Biochemistry* **2013**, *52*, 7559-7574, doi:10.1021/bi401138s.
39. Buelow, M.; Süßmuth, D.; Smith, L.D.; Aryani, O.; Castiglioni, C.; Stenzel, W.; Bertini, E.; Schuelke, M.; Knierim, E. Novel bi-allelic variants expand the SPTBN4-related genetic and phenotypic spectrum. *Eur J Hum Genet* **2021**, *29*, 1121-1128, doi:10.1038/s41431-021-00846-5.
40. Ortiz-Gonzalez, X.; Wierenga, K. SPTBN4 Disorder. In *GeneReviews(®)*, Adam, M.P., Everman, D.B., Mirzaa, G.M., Pagon, R.A., Wallace, S.E., Bean, L.J.H., Gripp, K.W., Amemiya, A., Eds.; University of Washington, Seattle
Copyright © 1993-2022, University of Washington, Seattle. GeneReviews is a registered trademark of the University of Washington, Seattle. All rights reserved.: Seattle (WA), 1993.

41. Nokelainen, P.; Peltoketo, H.; Vihko, R.; Vihko, P. Expression cloning of a novel estrogenic mouse 17 beta-hydroxysteroid dehydrogenase/17-ketosteroid reductase (m17HSD7), previously described as a prolactin receptor-associated protein (PRAP) in rat. *Mol Endocrinol* **1998**, *12*, 1048-1059, doi:10.1210/mend.12.7.0134.
42. Song, J.Y.; Ichtchenko, K.; Südhof, T.C.; Brose, N. Neuroligin 1 is a postsynaptic cell-adhesion molecule of excitatory synapses. *Proc Natl Acad Sci U S A* **1999**, *96*, 1100-1105, doi:10.1073/pnas.96.3.1100.
43. Ding, X.; Meng, S.; Zhou, J.; Yang, J.; Li, H.; Zhou, W. Translational Inhibition of α-Neurexin 2. *Sci Rep* **2020**, *10*, 3403, doi:10.1038/s41598-020-60289-8.
44. Gan, L.; Seki, A.; Shen, K.; Iyer, H.; Han, K.; Hayer, A.; Wollman, R.; Ge, X.; Lin, J.R.; Dey, G.; et al. The lysosomal GPCR-like protein GPR137B regulates Rag and mTORC1 localization and activity. *Nat Cell Biol* **2019**, *21*, 614-626, doi:10.1038/s41556-019-0321-6.
45. Visan, I. Compartmentalized selection. *Nature Immunology* **2016**, *17*, 1243-1243, doi:10.1038/ni.3595.
46. Bas, A.; Swamy, M.; Abeler-Dorner, L.; Williams, G.; Pang, D.J.; Barbee, S.D.; Hayday, A.C. Butyrophilin-like 1 encodes an enterocyte protein that selectively regulates functional interactions with T lymphocytes. *Proc Natl Acad Sci U S A* **2011**, *108*, 4376-4381, doi:10.1073/pnas.1010647108.
47. Gearing, D.P.; Thut, C.J.; VandeBos, T.; Gimpel, S.D.; Delaney, P.B.; King, J.; Price, V.; Cosman, D.; Beckmann, M.P. Leukemia inhibitory factor receptor is structurally related to the IL-6 signal transducer, gp130. *Embo j* **1991**, *10*, 2839-2848, doi:10.1002/j.1460-2075.1991.tb07833.x.
48. Dagoneau, N.; Scheffer, D.; Huber, C.; Al-Gazali, L.I.; Di Rocco, M.; Godard, A.; Martinovic, J.; Raas-Rothschild, A.; Sigaudy, S.; Unger, S.; et al. Null leukemia inhibitory factor receptor (LIFR) mutations in Stuve-Wiedemann/Schwartz-Jampel type 2 syndrome. *Am J Hum Genet* **2004**, *74*, 298-305, doi:10.1086/381715.
49. Hewitt, S.L.; Yin, B.; Ji, Y.; Chaumeil, J.; Marszalek, K.; Tenthorey, J.; Salvagiotto, G.; Steinel, N.; Ramsey, L.B.; Ghysdael, J.; et al. RAG-1 and ATM coordinate monoallelic recombination and nuclear positioning of immunoglobulin loci. *Nat Immunol* **2009**, *10*, 655-664, doi:10.1038/ni.1735.
50. Zhou, A.; Zhou, J.; Yang, L.; Liu, M.; Li, H.; Xu, S.; Han, M.; Zhang, J. A nuclear localized protein ZCCHC9 is expressed in cerebral cortex and suppresses the MAPK signal pathway. *J Genet Genomics* **2008**, *35*, 467-472, doi:10.1016/s1673-8527(08)60064-8.
51. Cui, X.; De Vivo, I.; Slany, R.; Miyamoto, A.; Firestein, R.; Cleary, M.L. Association of SET domain and myotubularin-related proteins modulates growth control. *Nat Genet* **1998**, *18*, 331-337, doi:10.1038/ng0498-331.

52. Blondeau, F.; Laporte, J.; Bodin, S.; Superti-Furga, G.; Payrastre, B.; Mandel, J.L. Myotubularin, a phosphatase deficient in myotubular myopathy, acts on phosphatidylinositol 3-kinase and phosphatidylinositol 3-phosphate pathway. *Hum Mol Genet* **2000**, *9*, 2223-2229, doi:10.1093/oxfordjournals.hmg.a018913.
53. Taylor, G.S.; Maehama, T.; Dixon, J.E. Myotubularin, a protein tyrosine phosphatase mutated in myotubular myopathy, dephosphorylates the lipid second messenger, phosphatidylinositol 3-phosphate. *Proc Natl Acad Sci U S A* **2000**, *97*, 8910-8915, doi:10.1073/pnas.160255697.
54. Schaletzky, J.; Dove, S.K.; Short, B.; Lorenzo, O.; Clague, M.J.; Barr, F.A. Phosphatidylinositol-5-phosphate activation and conserved substrate specificity of the myotubularin phosphatidylinositol 3-phosphatases. *Curr Biol* **2003**, *13*, 504-509, doi:10.1016/s0960-9822(03)00132-5.
55. Tsujita, K.; Itoh, T.; Ijuin, T.; Yamamoto, A.; Shisheva, A.; Laporte, J.; Takenawa, T. Myotubularin regulates the function of the late endosome through the gram domain-phosphatidylinositol 3,5-bisphosphate interaction. *J Biol Chem* **2004**, *279*, 13817-13824, doi:10.1074/jbc.M312294200.
56. Larsen, R.D.; Rajan, V.P.; Ruff, M.M.; Kukowska-Latallo, J.; Cummings, R.D.; Lowe, J.B. Isolation of a cDNA encoding a murine UDPgalactose:beta-D-galactosyl- 1,4-N-acetyl-D-glucosaminide alpha-1,3-galactosyltransferase: expression cloning by gene transfer. *Proc Natl Acad Sci U S A* **1989**, *86*, 8227-8231, doi:10.1073/pnas.86.21.8227.
57. Boucher, J.; Kleinridders, A.; Kahn, C.R. Insulin receptor signaling in normal and insulin-resistant states. *Cold Spring Harb Perspect Biol* **2014**, *6*, doi:10.1101/cshperspect.a009191.
58. Krüger, J.; Berger, C.; Weidle, K.; Schleinitz, D.; Tönjes, A.; Stumvoll, M.; Blüher, M.; Kovacs, P.; Klöting, N. Metabolic effects of genetic variation in the human REPIN1 gene. *International Journal of Obesity* **2019**, *43*, 821-831, doi:10.1038/s41366-018-0123-0.
59. Tawk, L.; Chicanne, G.; Dubremetz, J.-F.; Richard, V.; Payrastre, B.; Vial, H.J.; Roy, C.; Wengelnik, K. Phosphatidylinositol 3-Phosphate, an Essential Lipid in Plasmodium, Localizes to the Food Vacuole Membrane and the Apicoplast. *Eukaryotic Cell* **2010**, *9*, 1519-1530, doi:doi:10.1128/EC.00124-10.
60. Hao, H.X.; Cardon, C.M.; Swiatek, W.; Cooksey, R.C.; Smith, T.L.; Wilde, J.; Boudina, S.; Abel, E.D.; McClain, D.A.; Rutter, J. PAS kinase is required for normal cellular energy balance. *Proc Natl Acad Sci U S A* **2007**, *104*, 15466-15471, doi:10.1073/pnas.0705407104.

61. Soliz, J.; Soulage, C.; Borter, E.; van Patot, M.T.; Gassmann, M. Ventilatory responses to acute and chronic hypoxia are altered in female but not male Paskin-deficient mice. *Am J Physiol Regul Integr Comp Physiol* **2008**, *295*, R649-658, doi:10.1152/ajpregu.00876.2007.
62. Liu, P.; Torrens-Spence, M.P.; Ding, H.; Christensen, B.M.; Li, J. Mechanism of cysteine-dependent inactivation of aspartate/glutamate/cysteine sulfinic acid α-decarboxylases. *Amino Acids* **2013**, *44*, 391-404, doi:10.1007/s00726-012-1342-7.
63. Guion-Rain, M.C.; Portemer, C.; Chatagner, F. Rat liver cysteine sulfinate decarboxylase: purification, new appraisal of the molecular weight and determination of catalytic properties. *Biochim Biophys Acta* **1975**, *384*, 265-276, doi:10.1016/0005-2744(75)90115-1.
