# Supplementary figures and images for: Multi-Omics Analysis of Lung Tissue Demonstrates Changes to Lipid Metabolism during Allergic Sensitization in Mice
Source: Metabolites. 2023 Mar 9;13(3):406. doi: 10.3390/metabo13030406 (PMC10054742; doi:10.3390/metabo13030406)

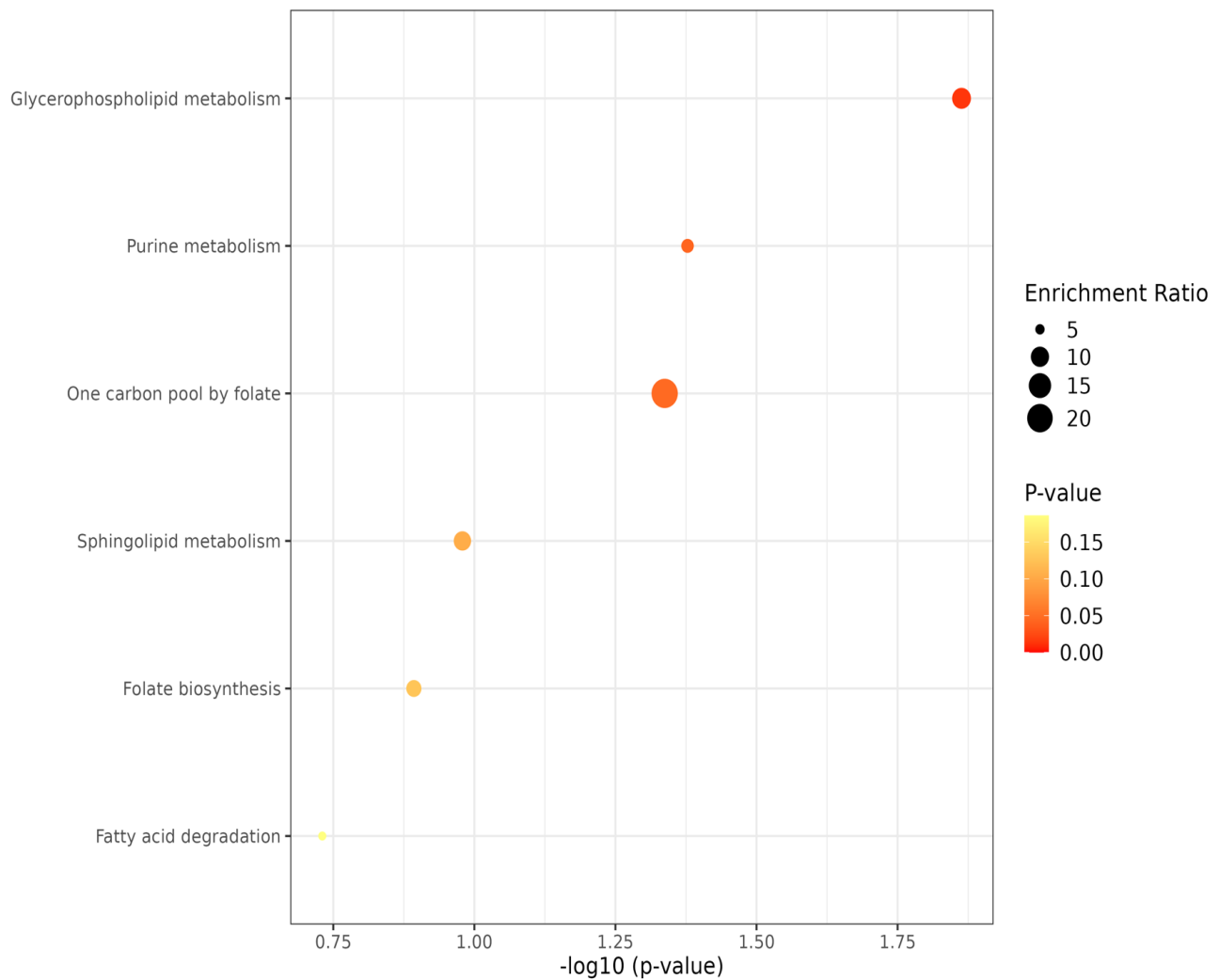

Supplement: Supplementary file 1 [file metabolites-13-00406-s001.zip › Fig_S1a.pdf]

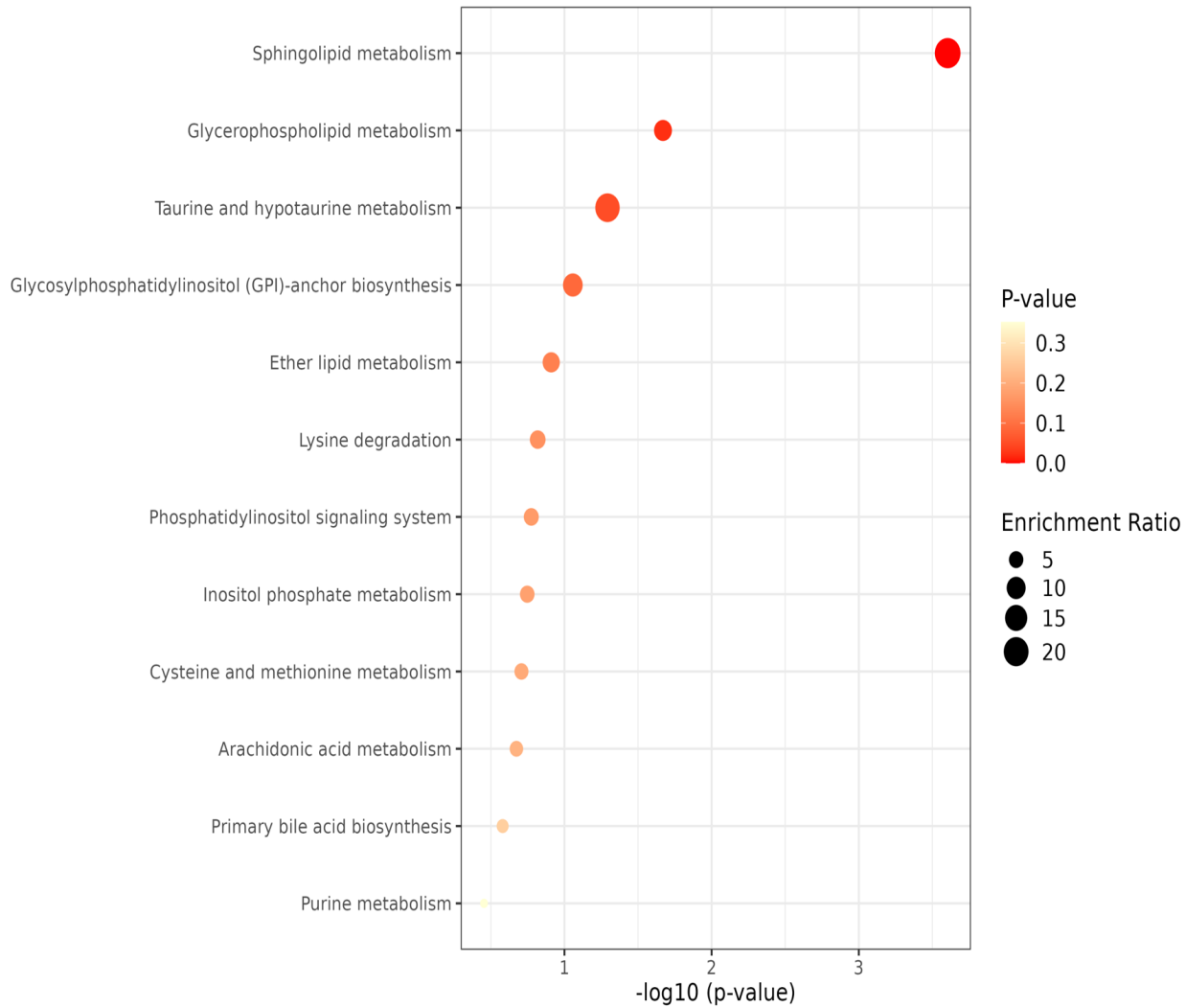

Supplement: Supplementary file 1 [file metabolites-13-00406-s001.zip › Fig_S1b.pdf]

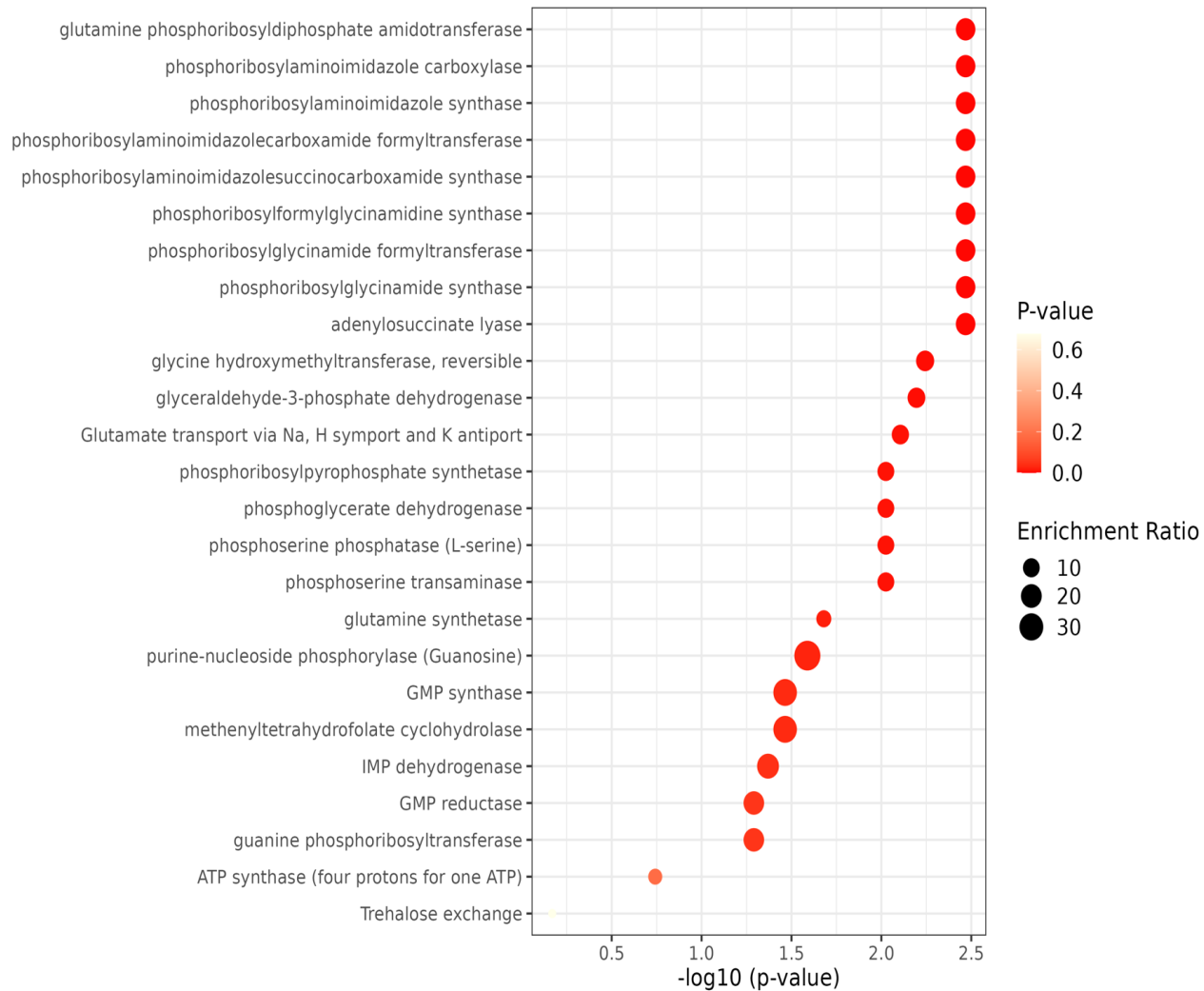

Supplement: Supplementary file 1 [file metabolites-13-00406-s001.zip › Fig_S2a.pdf]

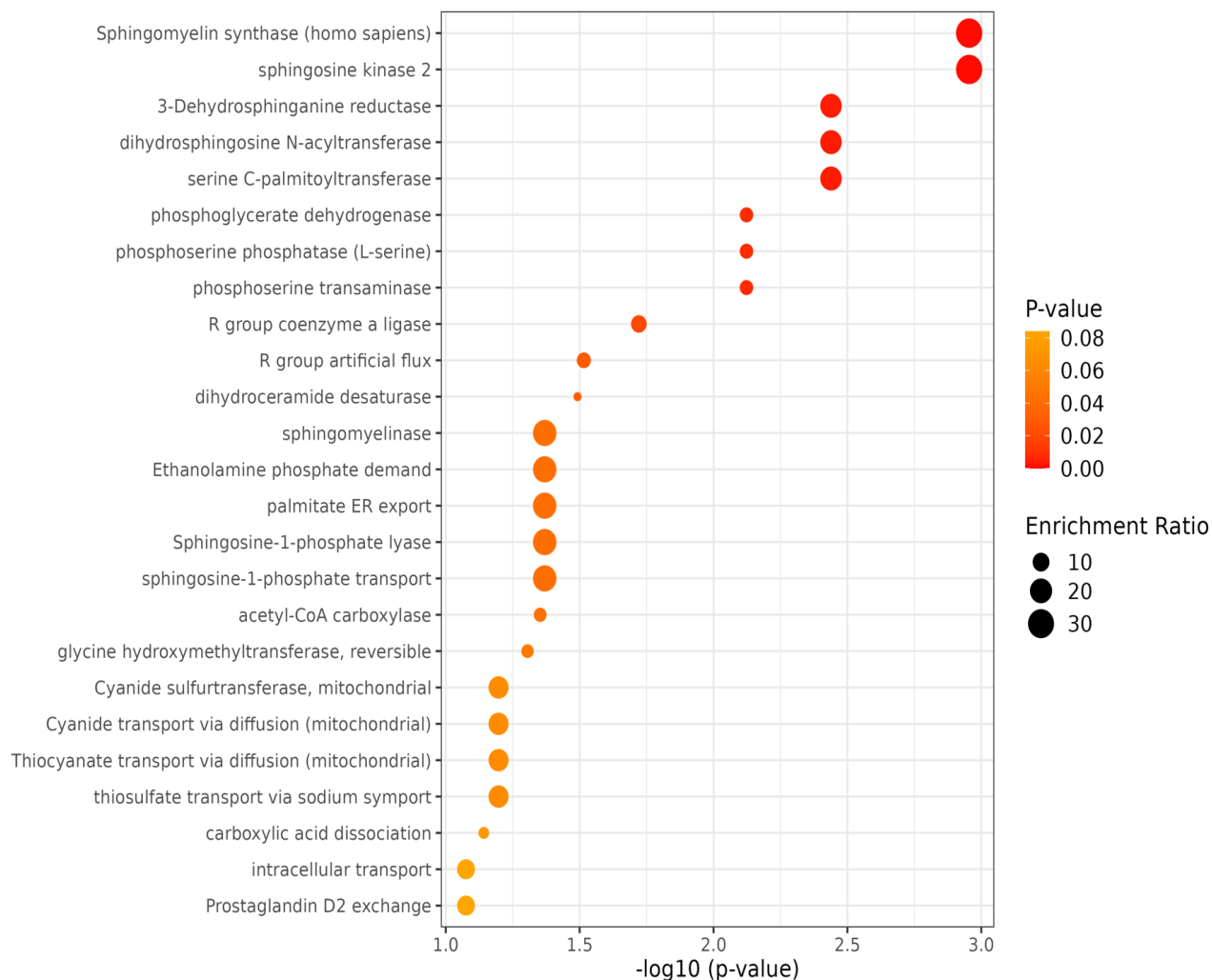

Supplement: Supplementary file 1 [file metabolites-13-00406-s001.zip › Fig_S2b.pdf]

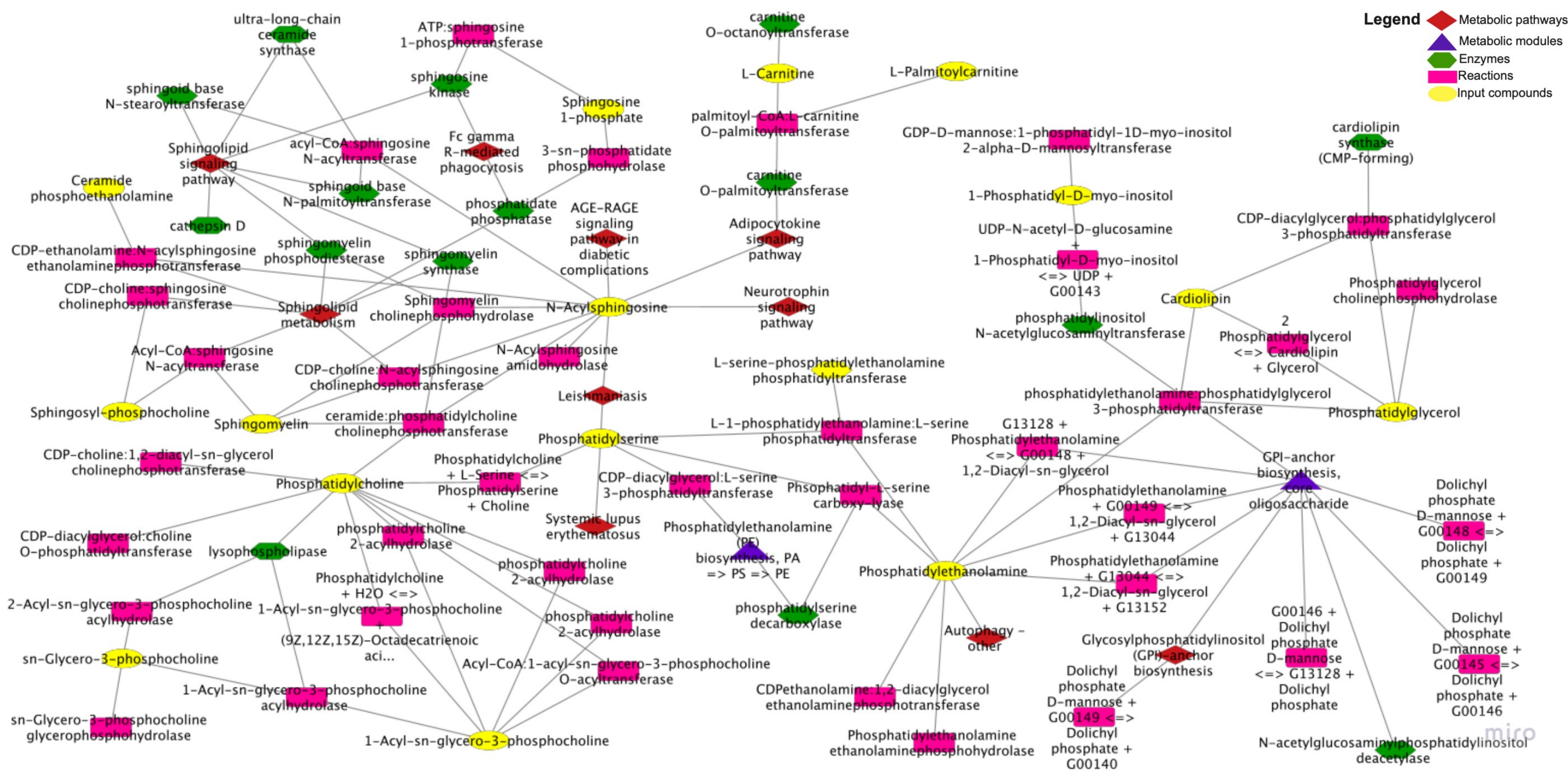

Supplement: Supplementary file 1 [file metabolites-13-00406-s001.zip › Fig_S3.pdf]

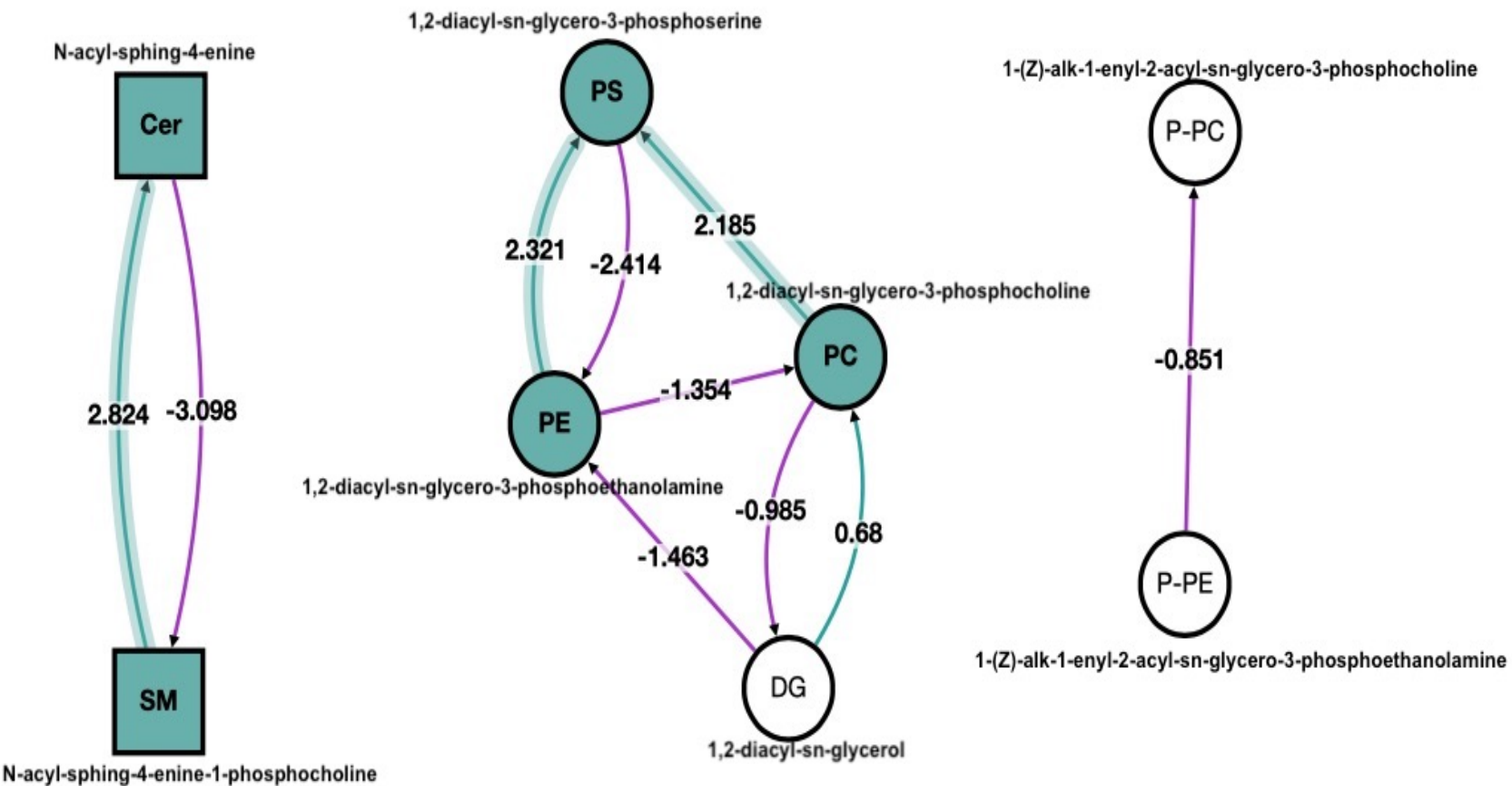

Nodes shape

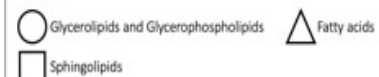

Nodes color

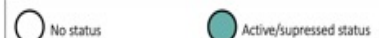

Edges color

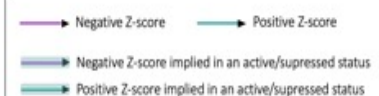

Supplement: Supplementary file 1 [file metabolites-13-00406-s001.zip › Fig_S4.pdf]

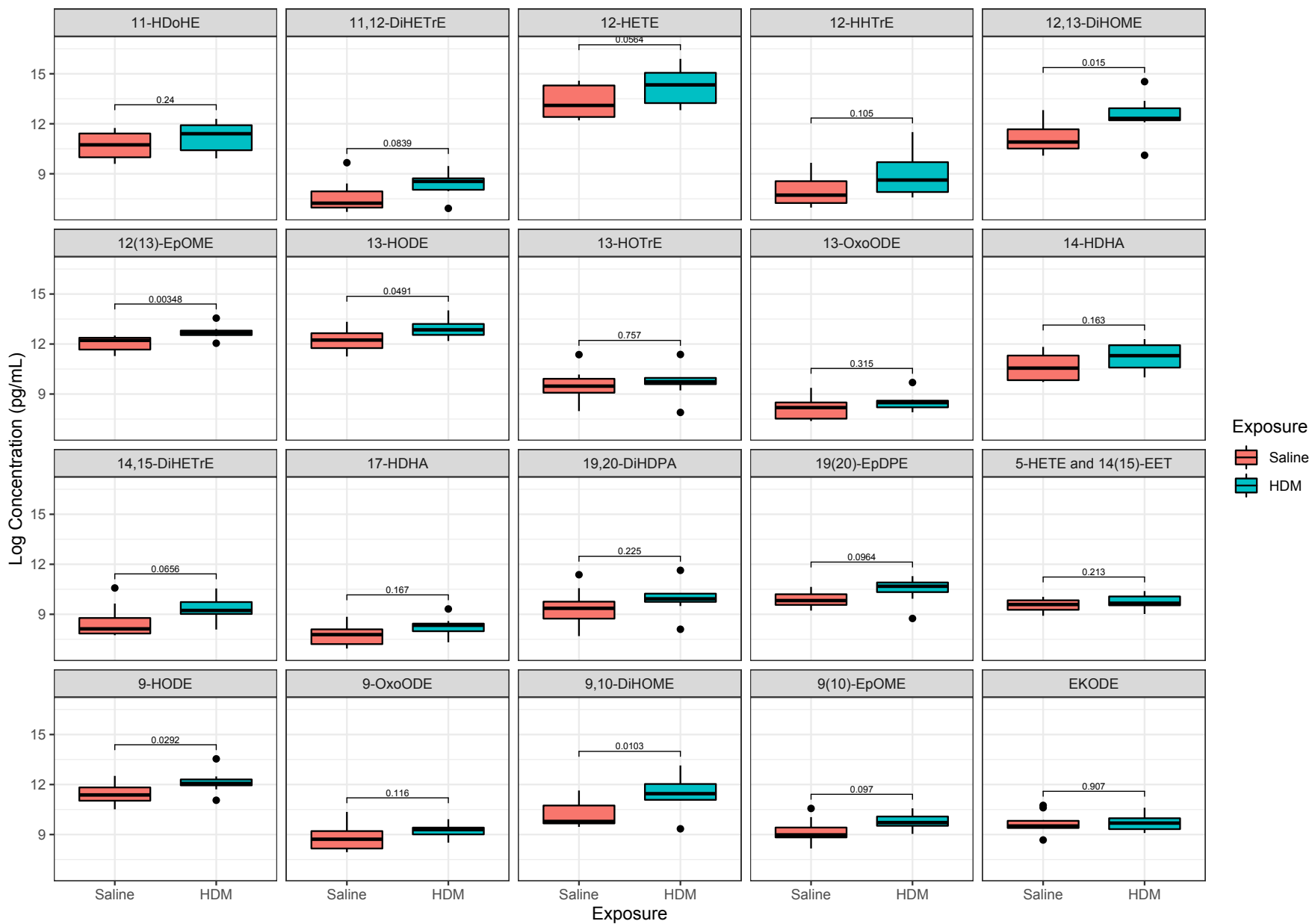

Supplement: Supplementary file 1 [file metabolites-13-00406-s001.zip › Fig_S5.pdf]

Log Concentration (pg/mg)

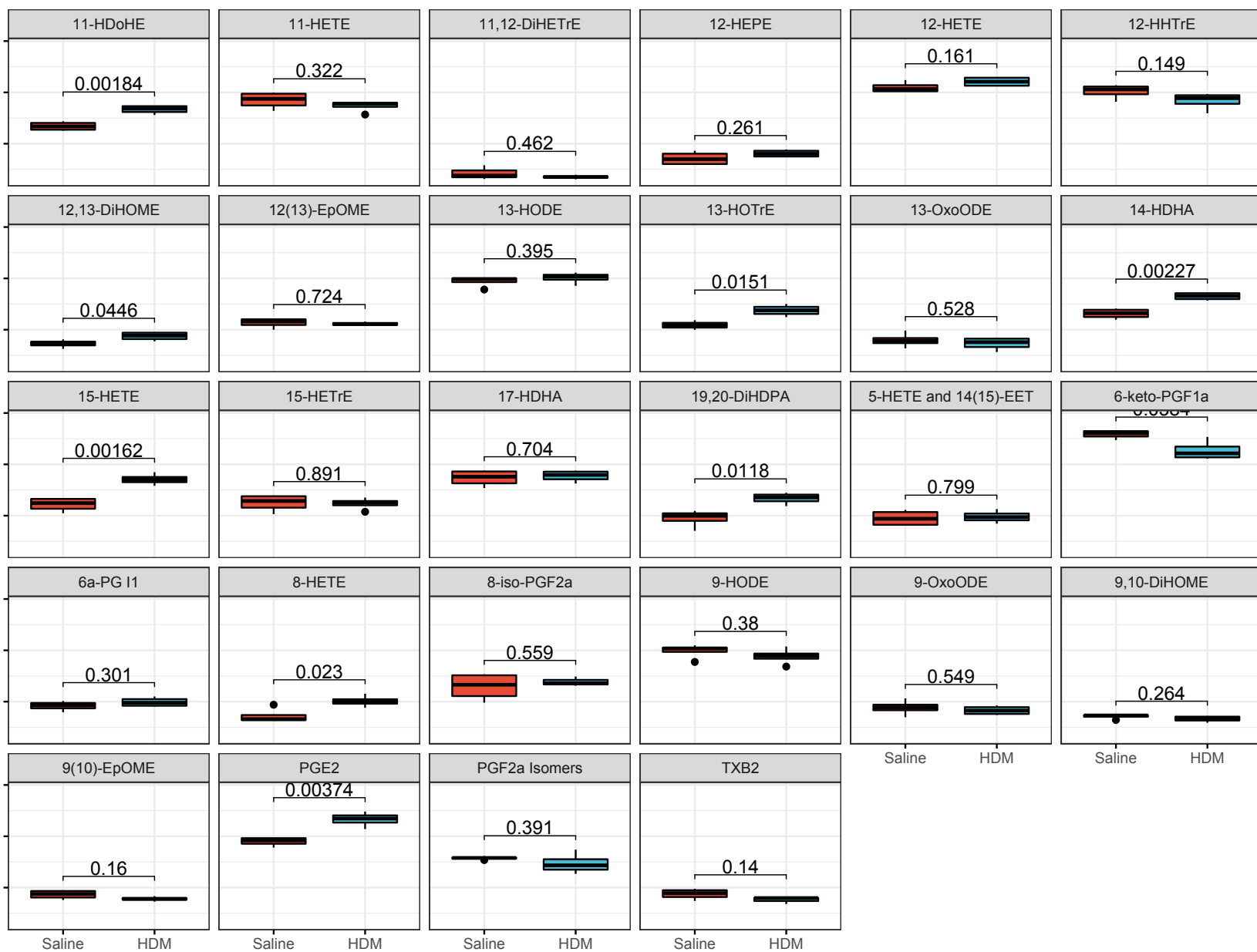

Supplement: Supplementary file 1 [file metabolites-13-00406-s001.zip › Fig_S6.pdf]

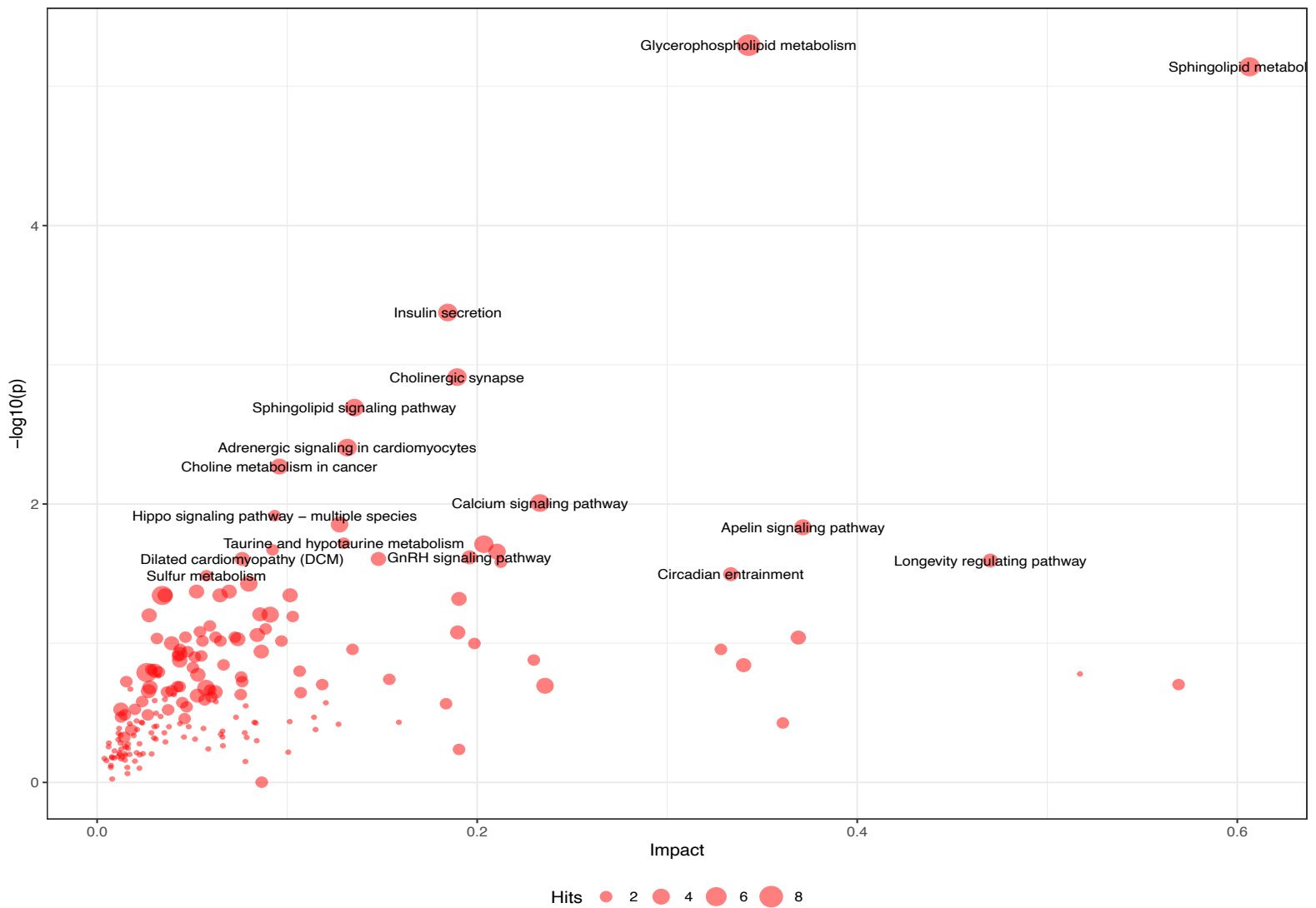

Supplement: Supplementary file 1 [file metabolites-13-00406-s001.zip › Fig_S7.pdf]
